# Supplementary material for: Layered van der Waals crystals with hyperbolic light dispersion
Source: Nat Commun. 2017 Aug 22;8:320. doi: 10.1038/s41467-017-00412-y (PMC5567251; doi:10.1038/s41467-017-00412-y)
Supplement: Supplementary file 1 — Supplementary Information [file 41467_2017_412_MOESM1_ESM.pdf]

Description of Supplementary Files

File Name: Supplementary Information
Description: Supplementary Figures, Supplementary Notes and Supplementary References

File Name: Peer Review File

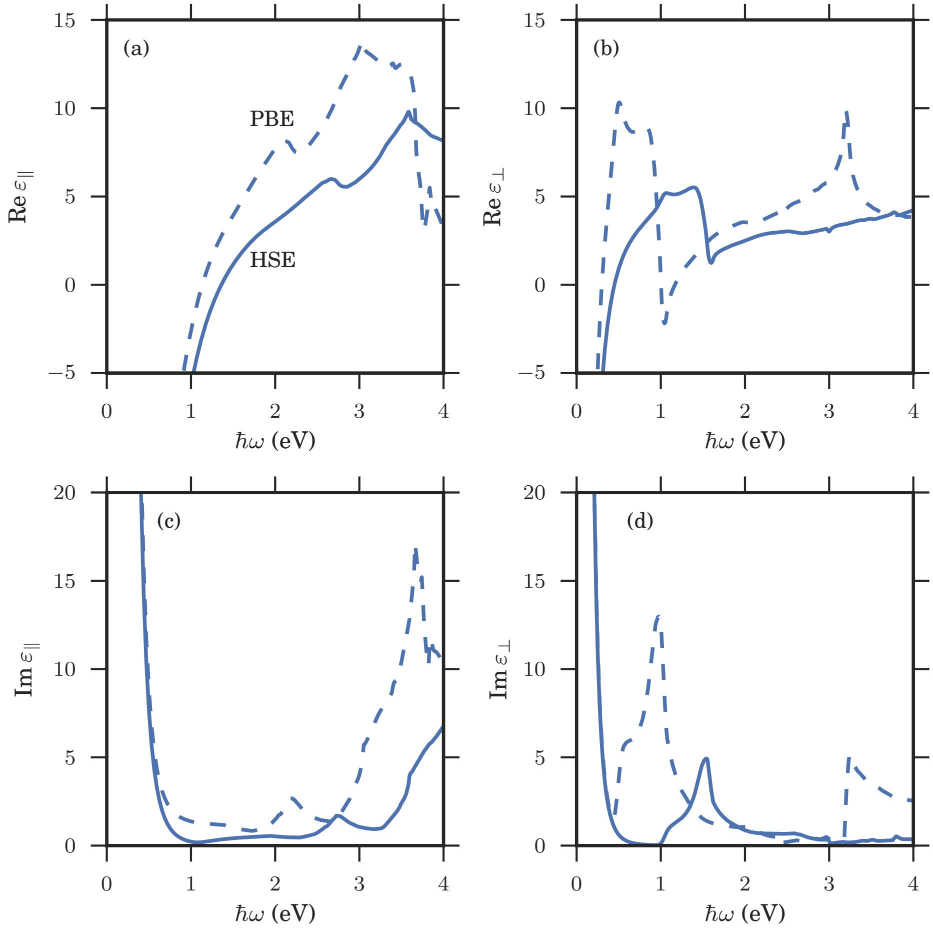

SUPPLEMENTARY FIGURE 1. **Comparison of the dielectric functions calculated with HSE and PBE for 2H-$\mathbf{TaS_2}$** Calculated dielectric tensor of 2H-TaS_2 based on PBE (full line) and HSE (dashed line) wave functions and energies. Real (a) and imaginary (c) parts of the in-plane component of the dielectric tensor and real (b) and imaginary (d) parts of the out-of-plane component of the dielectric tensor. As explained in the methods section the effect of the HSE functional is applied as a scissor operator on top of the PBE eigenvalues shifting all fully occupied bands by -0.55 eV and all unoccupied bands by 0.55 eV. Considering first the imaginary part (c) and (d), the main effect is seen to be the increasing of the interband transitions with 0.55 eV. This is most clear for the out-of-plane component (d). The increased interband transition reduces the screening of the plasmafrequencies and the effect on the real part of the dielectric functions (a) and (b) is seen to be an increase of the plasmafrequencies, both in-plane and out-of-plane. These results mean that we should consider the results for the hyperbolic regions in Figure 1 to be shifted towards higher frequencies in reality.

SUPPLEMENTARY NOTE 1: EFFECTS OF SPATIAL DISPERSION

For natural hyperbolic materials the Purcell factor is limited either by the distance of the dipole to the surface (h) or by spatial dispersion effects. Below we investigate the relative importance of both. The essential effect of spatial dispersion is to introduce a wave-vector dependence of the dielectric function. To lowest order this can be modelled through a simple modification of Drude theory[1]

$$\varepsilon_{\perp}(\omega, k) = 1 - \frac{\omega_{\mathrm{p},\perp}^2}{\omega(\omega + i\eta)} \tag{1}$$

$$\varepsilon_{\parallel}(\omega, k) = 1 - \frac{\omega_{\mathrm{p},\parallel}^2}{\omega(\omega + i\eta) - \beta^2 k^2}, \tag{2}$$

where ω_{p} is the bulk plasmafrequency, η is the electronic relaxation rate, $\beta^2 = (3/5)v_{\mathrm{F}}^2$ is a non-local parameter describing the strength of the non-local effects where v_{F} is the in-plane Fermi velocity and $\parallel$ and $\perp$ respectively mark the in-plane and out-of-plane components. The effect of spatial dispersion on the Purcell factor for 2H-TaS_2 has been determined using this wave vector dependence for the intraband contribution to the dielectric function. The non-local effects are included only in the in-plane component since the Fermi velocity out-of-plane is small due to the small hybridization. The Fermi-velocity was found to be 3×10^5 ms^{-1} for 2H-TaS_2 determined by investigating its bandstructure. Supplementary Figure 2(a) shows the dielectric function of 2H-TaS_2 (HSE) for reference. The effect of spatial dispersion on the Fresnel reflection coefficient r_{p}, shown in Supplementary Figure 2(b), is to introduce a cutoff at $\hbar\omega = \beta k$ which otherwise would be finite for $k_{\parallel} \to \infty$ without spatial dispersion effects[1]. Spatial dispersion also manifest in the Purcell factor when the dipole emitter is positioned at very small distances to the surface. This is evident for emission enhanced by metallic spheres[2], but it applies to other geometries as well. Here, the emitted evanescent field of the point dipole must reach the material for the spatial dispersion effects to be important, see Eq. (2). This cutoff happens at a wave-vector of $k_{\mathrm{c}} = \pi/h$ shown by the vertical dashed line which must be larger than the characteristic non-local wavevector $k_{\mathrm{nl}} = \omega/\beta$ for spatial dispersion effects to be important. It is evident from Supplementary Figure 2(c), showing the calculated Purcell factor in the local and non-local approximations, that the effect of spatial dispersion on the Purcell factor is negligible when the dipole is placed a distance of $h = 1$ nm from the metamaterial (Supplementary Figure 2(c)). For simplicity, we may thus safely ignore aspects of spatial dispersion.

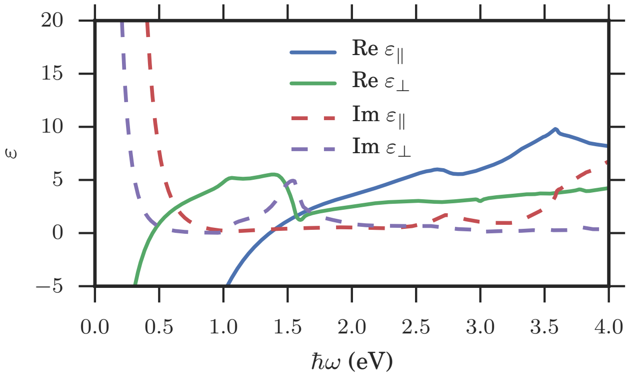

(a)

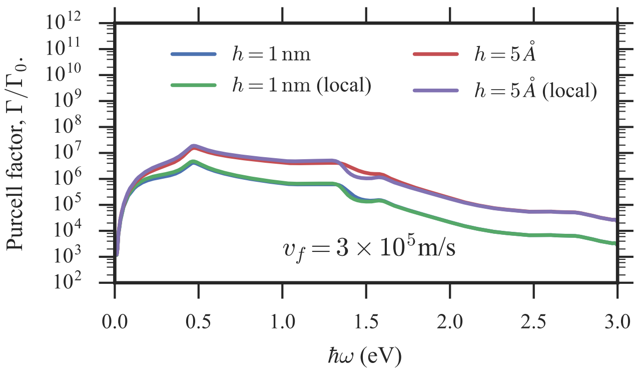

(c)

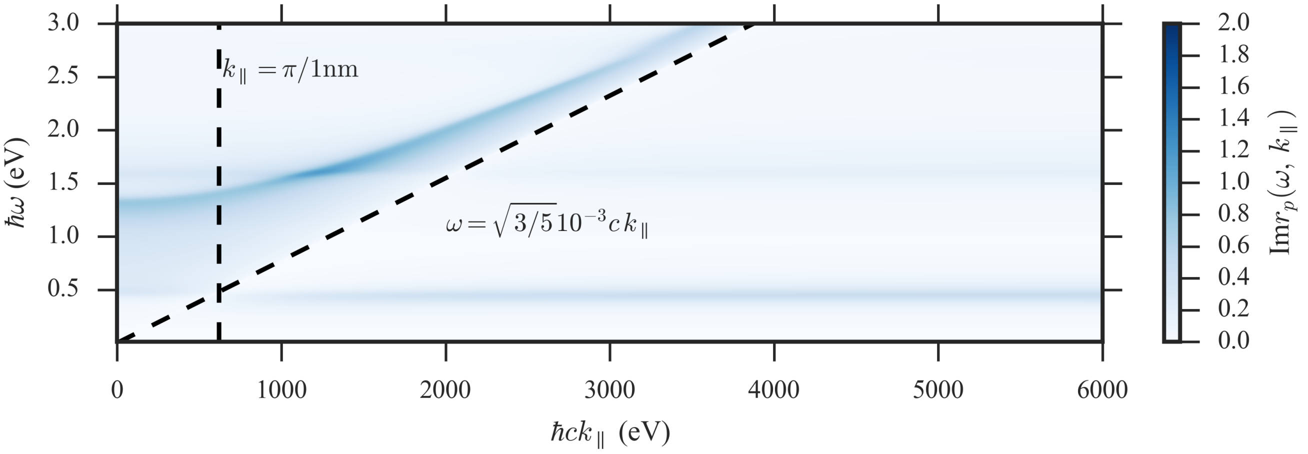

(b)

SUPPLEMENTARY FIGURE 2. **Effect of spatial dispersion on the Purcell factor of 2H-TaS$_2$ (HSE)** (a) The dielectric function of 2H-TaS$_2$ (HSE) for $k = 0$. Real part of the dielectric functions are shown by full lines (blue: in-plane component, green: out-of-plane component) and dashed line show the imaginary parts (red: in-plane component, violet: out-of-plane component). (b) The Fresnell reflection coefficient r_p in the momentum-frequency plane. (c) The Purcell factor calculated with and without spatial dispersion for varying distance between the dipole and the metamaterial substrate.

SUPPLEMENTARY NOTE 2: VALIDITY OF EFFECTIVE MEDIUM THEORY FOR PREDICTING HYPERBOLIC REGIMES.

Our calculations for the van der Waals heterostructures reported in Figures 4 and 5 were based on effective medium theory (EMT),

$$\varepsilon_{\mathrm{eff},\parallel} = f_1 \varepsilon_{1,\parallel} + (1 - f_1)\varepsilon_{2,\parallel} \tag{3}$$

$$\frac{1}{\varepsilon_{\mathrm{eff},\perp}} = \frac{f_1}{\varepsilon_{1,\perp}} + \frac{1 - f_1}{\varepsilon_{2,\perp}} \tag{4}$$

where f_1 $(1 - f_1)$ is the fill fraction of component 1 (2) with dielectric function ε_1 (ε_2) and $\parallel$ and $\perp$ mark the in-plane and out-of-plane components of the dielectric function. There are in fact two different approximations behind this approach. First, since EMT homogenises the material, reflection of electromagnetic waves at the internal interfaces of the heterostructure are neglected. In Ref. 3 we showed, using graphene/hBN heterostructures as an example, that the effect of reflections becomes important only for periods larger than ~ 60 monolayers. The second approximation is that below this limit, where the assumption of a homogeneous optical material is indeed valid, the true macroscopic dielectric function of the heterostructure can be obtained from EMT. Below we show, again using graphene/hBN heterostructures as an example, that EMT gives a decent description of the dielectric constant for heterostructures with periods larger than ~ 20 monolayers, while an excellent prediction of the hyperbolic frequency range is obtained for any heterostructure even the thinnest consisting of alternating graphene and hBN monolayers. The graphene/hBN heterostructure was chosen for computational simplicity, but we expect that the conclusions apply to general van der Waals heterostructures.

In Supplementary Figure 3(a) we compare the ab-initio calculated macroscopic dielectric constants of graphene/hBN heterostructures of varying periods with the EMT result. To achieve a metallic in-plane response the structures were doped corresponding to a Fermi level of 0.5 eV above the Dirac point. The EMT result is obtained from the ab-initio calculated dielectric constants of bulk hBN and (doped) graphite, respectively. Any deviation from EMT is due to hybridization between hBN and graphene at the interfaces. It can be seen that EMT accurately reproduces the hyperbolic frequency regime (ending where $\mathrm{Re}\epsilon(\omega) = 0$) for all the heterostructures. This is due to the fact that the in-plane dielectric function is well described by EMT for all heterostructures and that the out-of-plane component remain positive. The comparably worse description of the out-of-plane component by EMT to the in-plane component, is due to the rather significant interface scattering for electrons moving perpendicular to the interface. We note that $1/N$ (N being the period) extrapolation of the first-principles results coincide almost exactly with the EMT result consistent with the view that EMT neglects interface scattering. It is unclear whether the difference in the electronic structure between graphene and the TMDs would change any of these conclusions. A similar analysis have therefore been performed on 2H-TaS_2 with vacuum substituted for the insulating material. Vacuum was necessary since no other material in this study matches the lattice constant of 2H-TaS_2. The results are shown in Supplementary Figure 3(b) and it is clear the the conclusions remain valid.

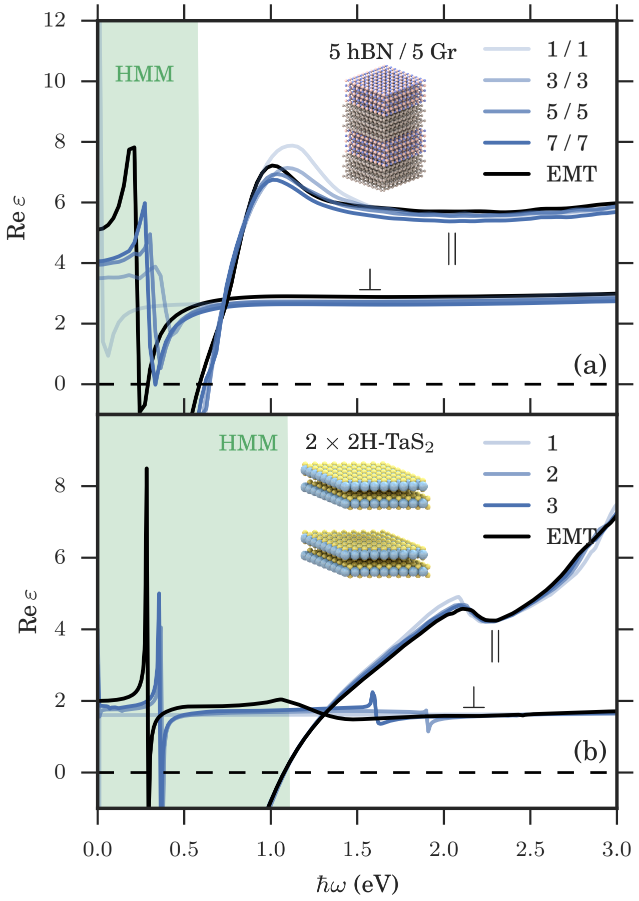

SUPPLEMENTARY FIGURE 3. **Effective medium theory for the dielectric function tested from first principles** Effective medium theory for the dielectric function is tested from first principles by (a) assembling heterostuctures composed graphene and hexagonal boron-nitride and (b) by analyzing the dielectric properties of multilayered 2H-TaS_2.

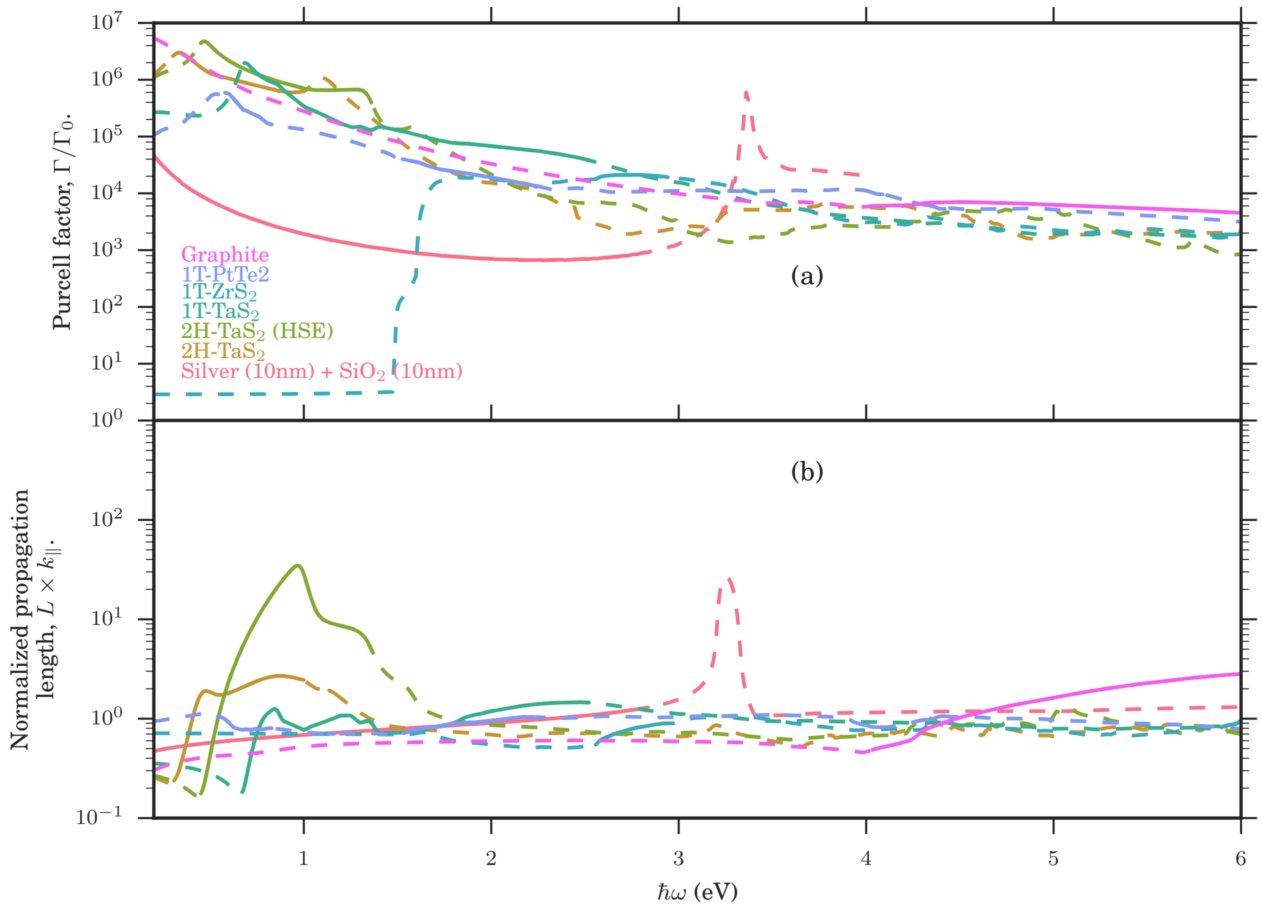

SUPPLEMENTARY FIGURE 4. **Calculated Purcell factors for metallic and semi-conducting TMDs** (a) Purcell factors and (b) propagation lengths for some of the naturally hyperbolic materials compared with the silver-Si_2 metamaterial. Only one semi-conducting hyperbolic material has been included (1T-ZrS_2) since all of them behave similarly above their interband onset. A full lines mark the hyperbolic regimes (as predicted by EMT for the metamaterial) and dashed lines are drawn if the materials are not hyperbolic. We emphasize that the metallic natural hyperbolic materials show Purcell factors much larger than both the metamaterials and the semi-conducting hyperbolic materials. The calculated propagation lengths also highlight the importance of the special bandstructure of 2H-TaS_2, by comparing the PBE results with the HSE, showing that the larger bandgaps entail longer propagation lengths.

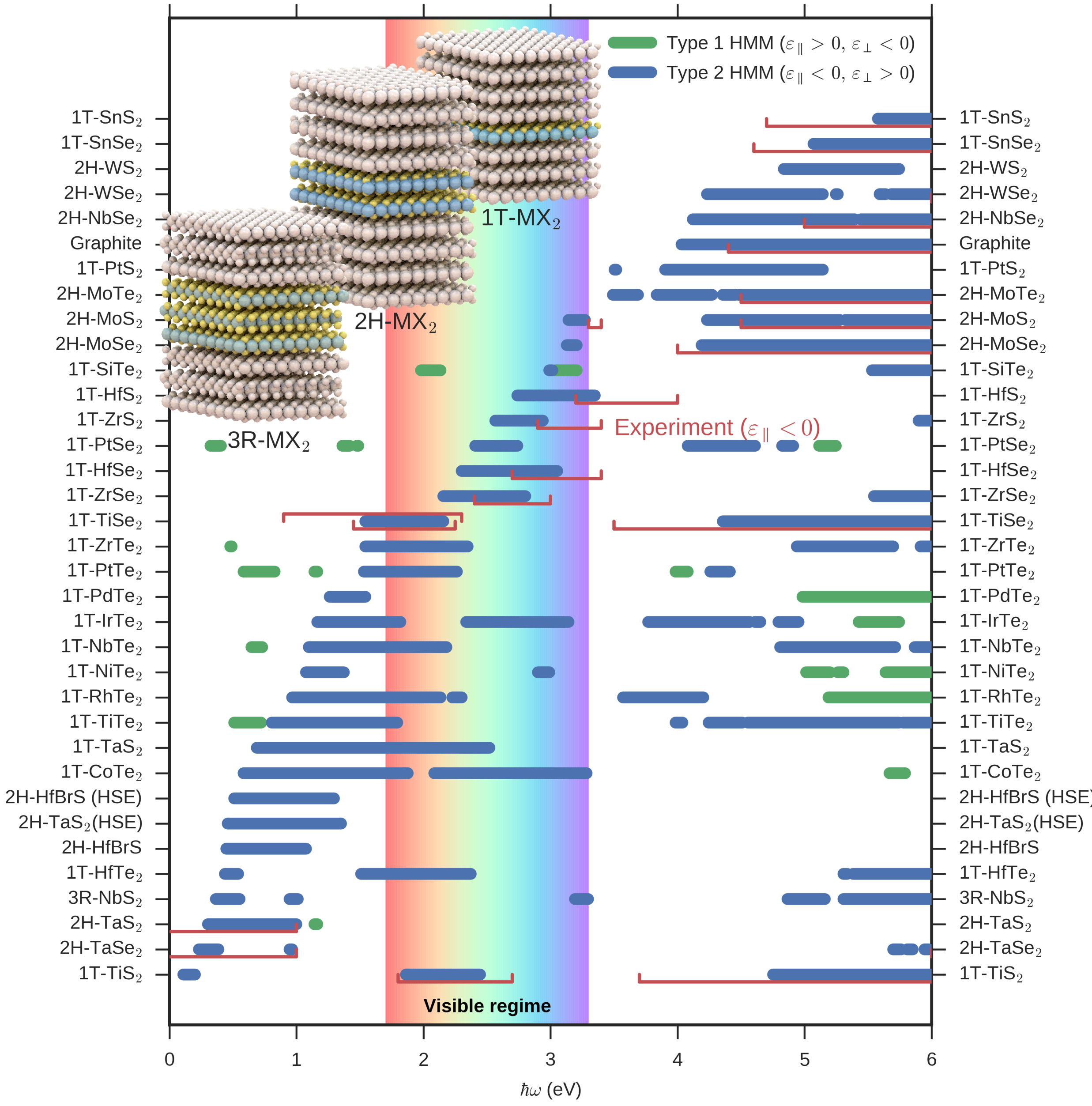

SUPPLEMENTARY FIGURE 5. **Hyperbolic regimes compared with experiments.** Hyperbolic regimes like Fig. 2, but compared with experimental data where $\varepsilon_{\parallel} < 0$ which is a good indicator for hyperbolic properties. Experimental data references: 2H-NbSe$_2$[4], Graphite[5], 2H-MoTe$_2$[6], 2H-MoS$_2$[6, 7], 2H-MoSe$_2$[6], 1T-ZrS$_2$[8], 1T-ZrSe$_2$[9], 1T-HfSe$_2$[9], 1T-HfS$_2$[9], 1T-TiSe$_2$[9, 10], 2H-TaS$_2$[4], 2H-TaSe$_2$[4], 1T-TiS$_2$[9], 1T-SnS$_2$[11], 1T-SnSe$_2$[11].

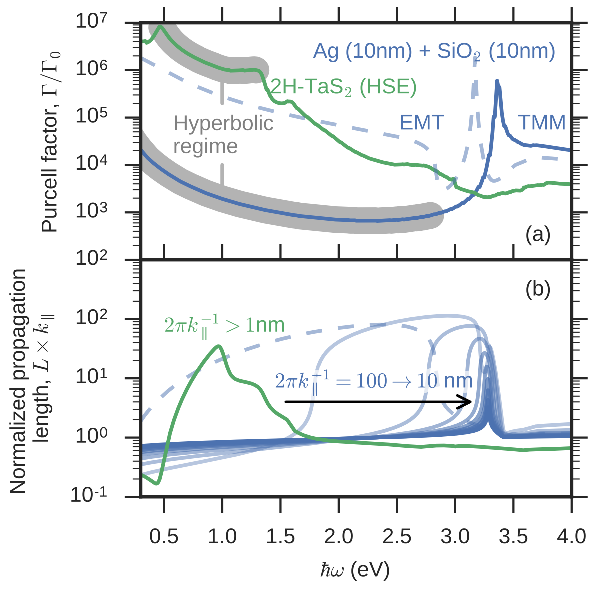

SUPPLEMENTARY FIGURE 6. **EMT vs TMM** This figure shows results of Fig. 4 compared to the corresponding results of effective medium theory (EMT, dashed lines). There is no change to the results for 2H-TaS_2 due to the lack of internal structure. In contrast, EMT predicts a large broadband response for the Ag/SiO_2 metamaterial compared to the transfer matrix method (TMM). This difference is due to the loss of the hyperbolic response due to the vanishing overlap over surface plasmons on the Ag/SiO_2 interfaces.

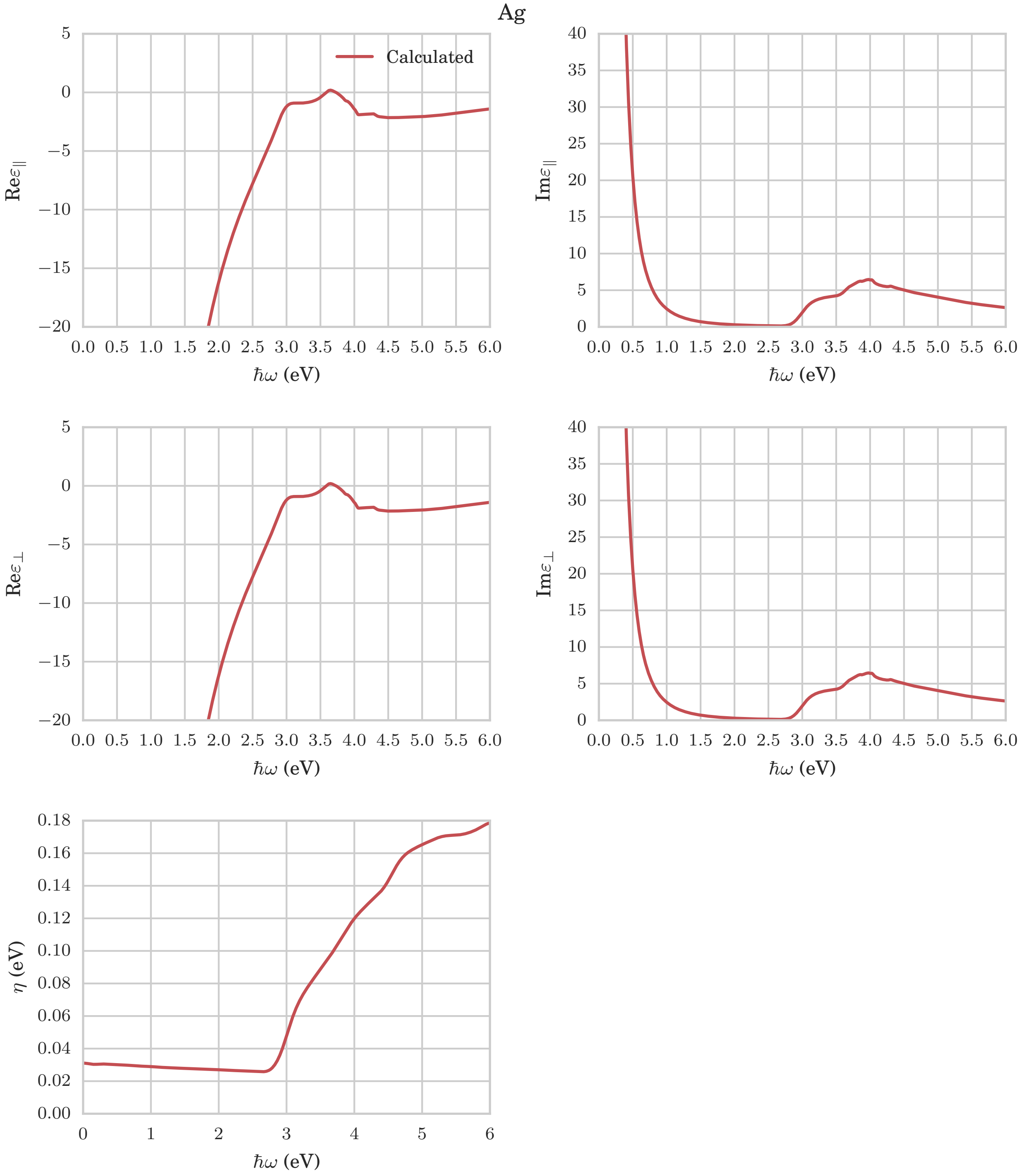

SUPPLEMENTARY FIGURE 7. **Calculated dielectric function of Ag** In-plane and out-of-plane components of the calculated dielectric function of silver. Silver is isotropic so there is no difference between different components of the dielectric tensor. The Drude scattering rate η is also shown.

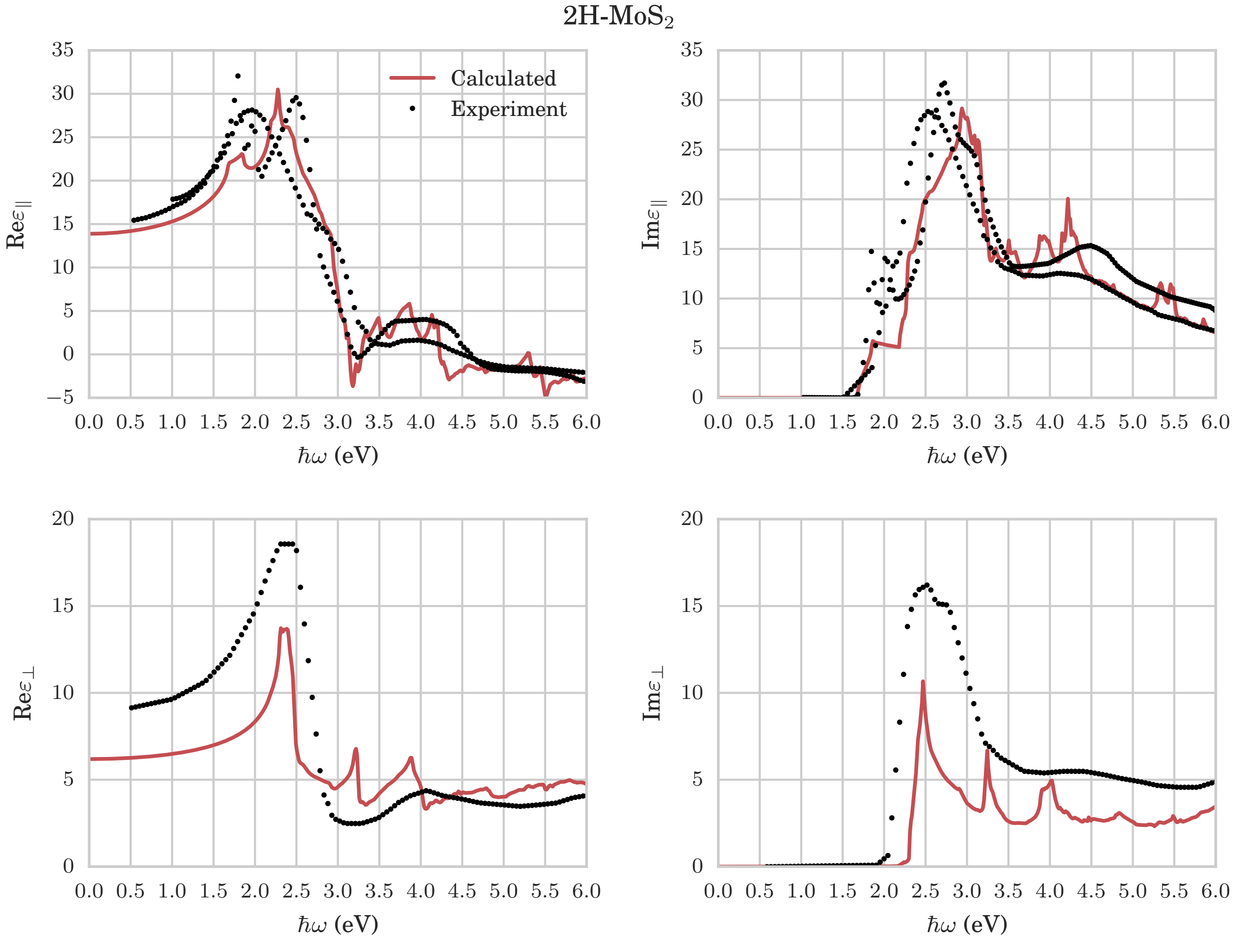

SUPPLEMENTARY FIGURE 8. **Calculated dielectric function of 2H-MoS_2** Calculated in-plane and out-of-plane components of the dielectric tensor of 2H-MoS_2. Experimental data from [6, 7].

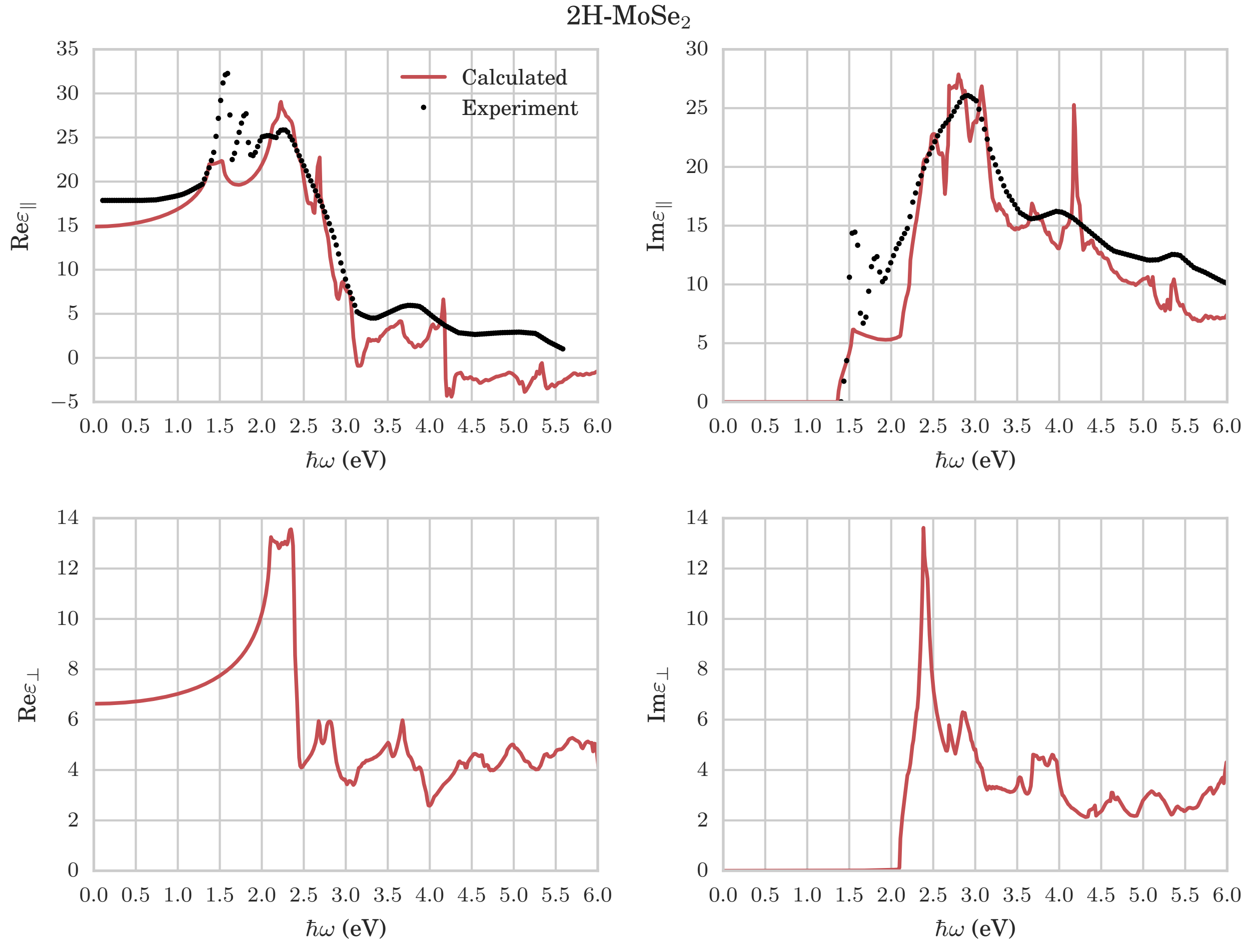

SUPPLEMENTARY FIGURE 9. **Calculated dielectric function of 2H-MoSe$_2$** Calculated in-plane and out-of-plane components of the dielectric tensor of 2H-MoSe$_2$. Experimental data from [6].

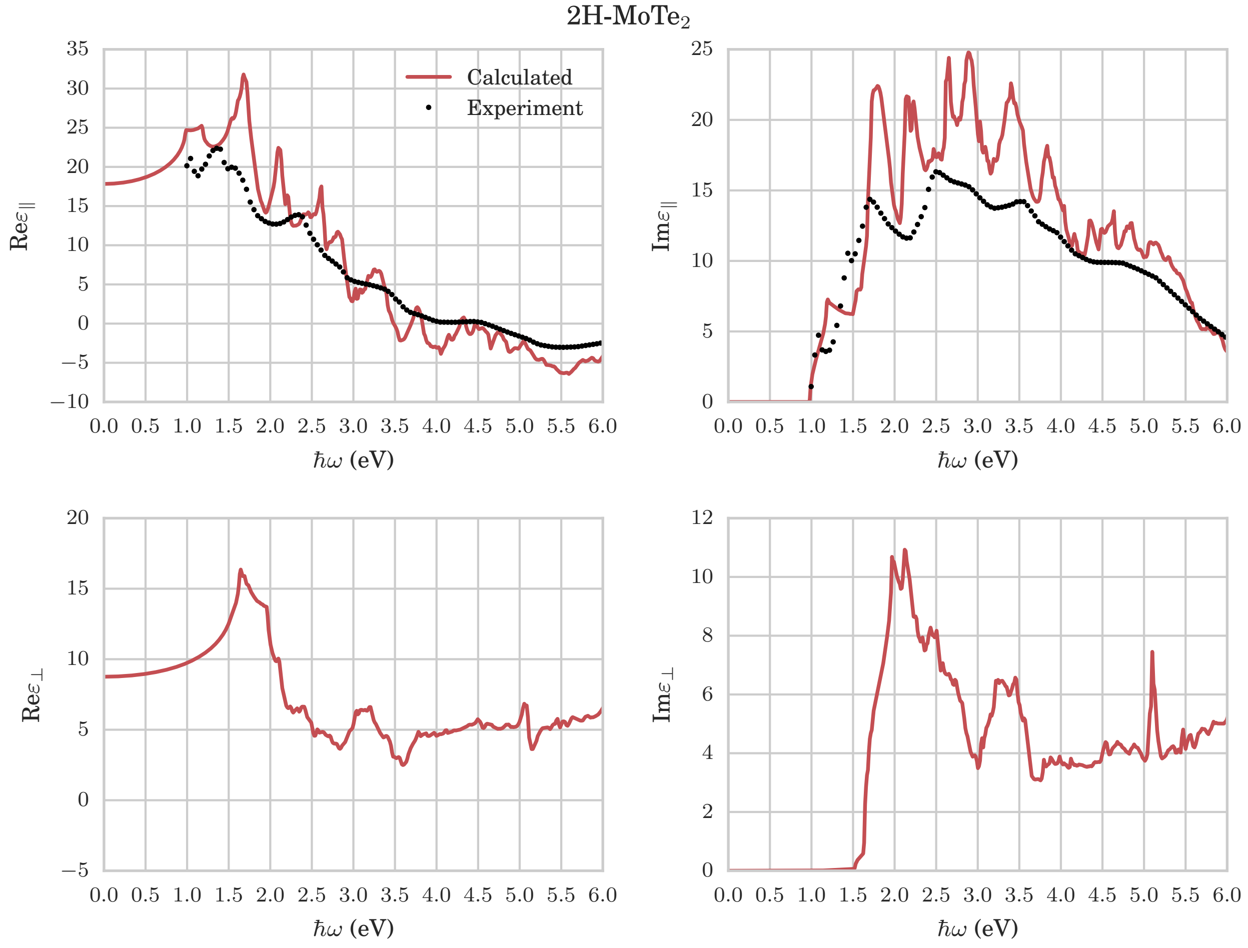

SUPPLEMENTARY FIGURE 10. **Calculated dielectric function of 2H-MoTe$_2$** Calculated in-plane and out-of-plane components of the dielectric tensor of 2H-MoTe$_2$. Experimental data from [6].

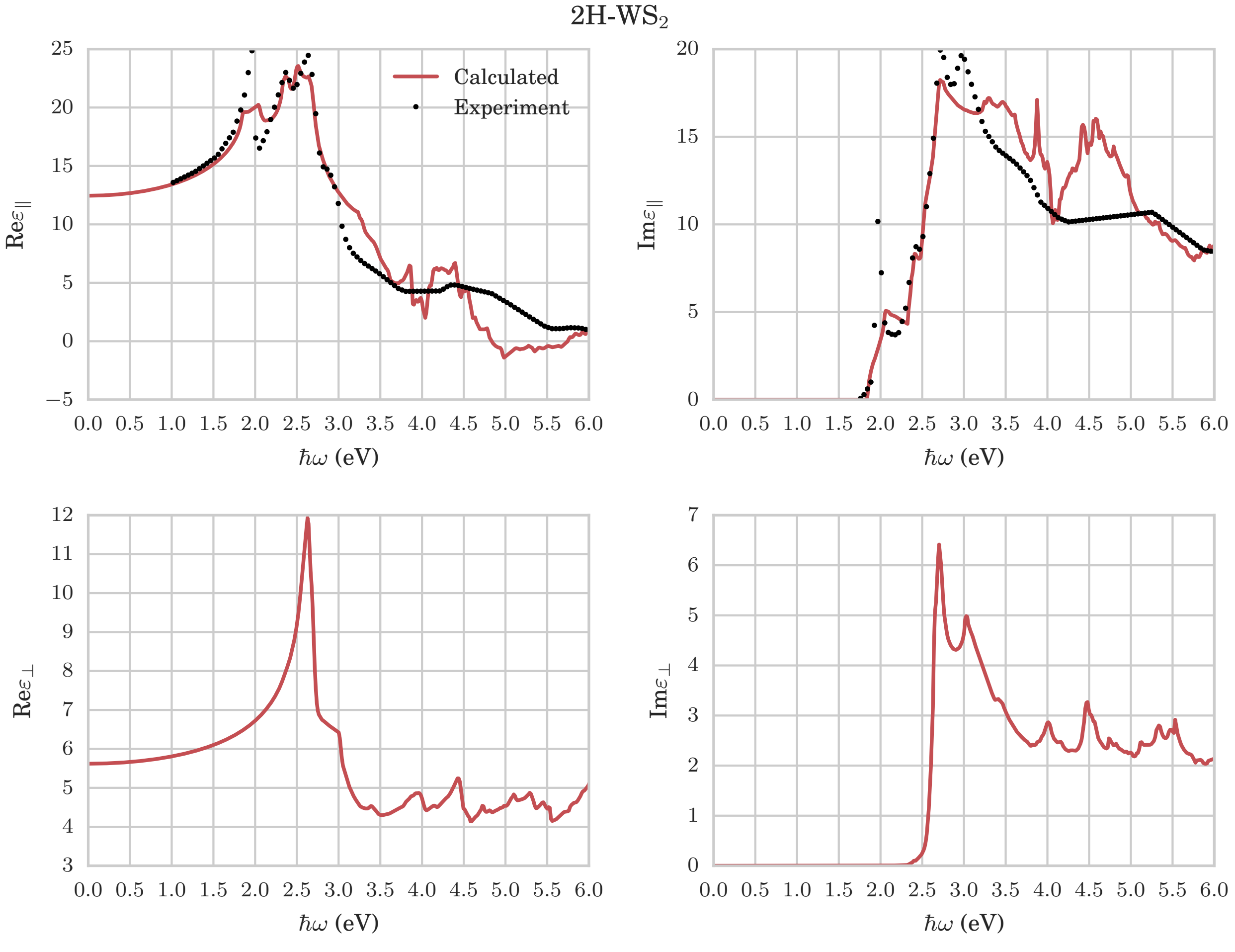

SUPPLEMENTARY FIGURE 11. **Calculated dielectric function of 2H-WS$_2$** Calculated in-plane and out-of-plane components of the dielectric tensor of 2H-WS$_2$. Experimental data from [12].

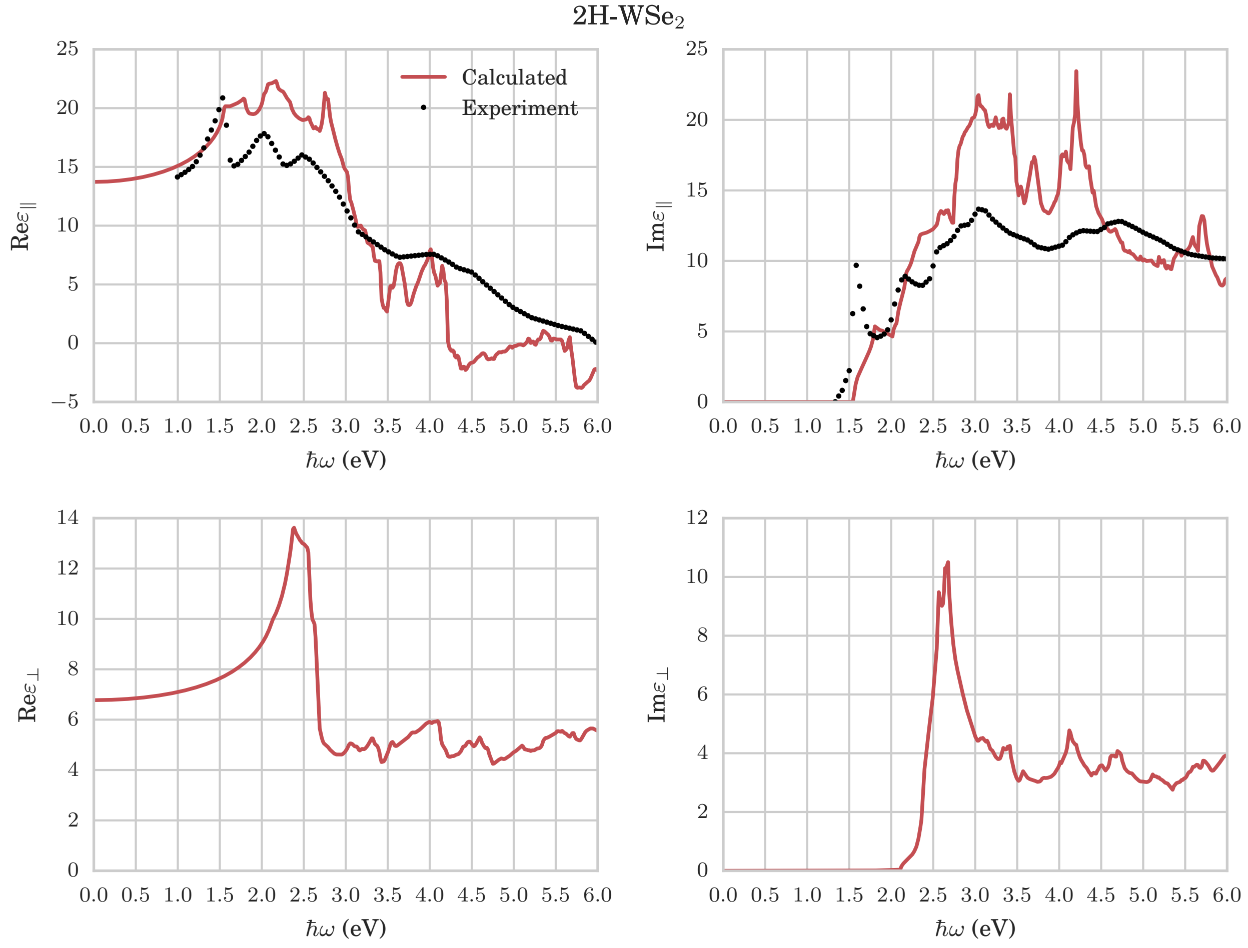

SUPPLEMENTARY FIGURE 12. **Calculated dielectric function of 2H-WSe$_2$** Calculated in-plane and out-of-plane components of the dielectric tensor of 2H-WSe$_2$. Experimental data from [12].

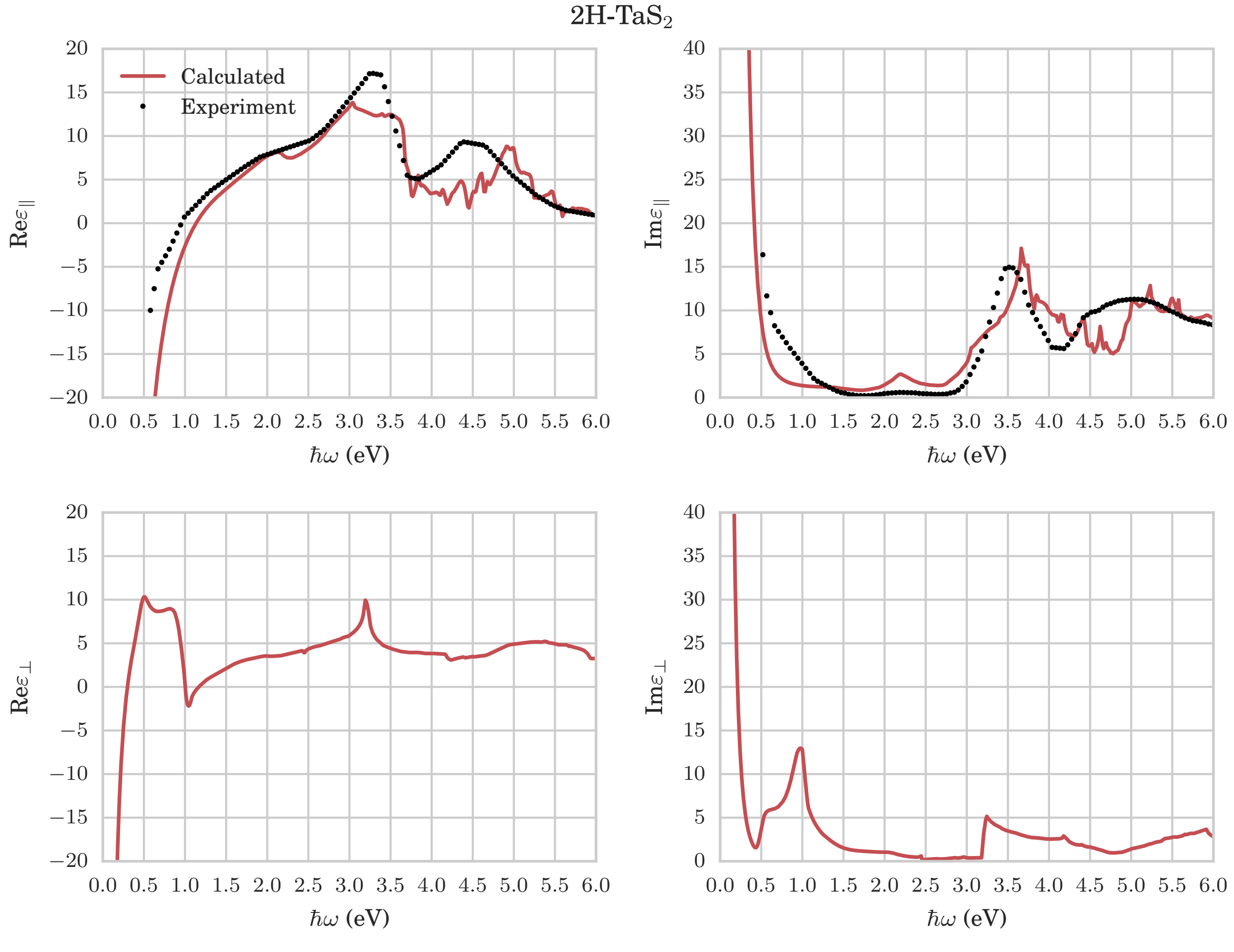

SUPPLEMENTARY FIGURE 13. **Calculated dielectric function of 2H-TaS$_2$** Calculated in-plane and out-of-plane components of the dielectric tensor of 2H-TaS$_2$. Experimental data from [4].

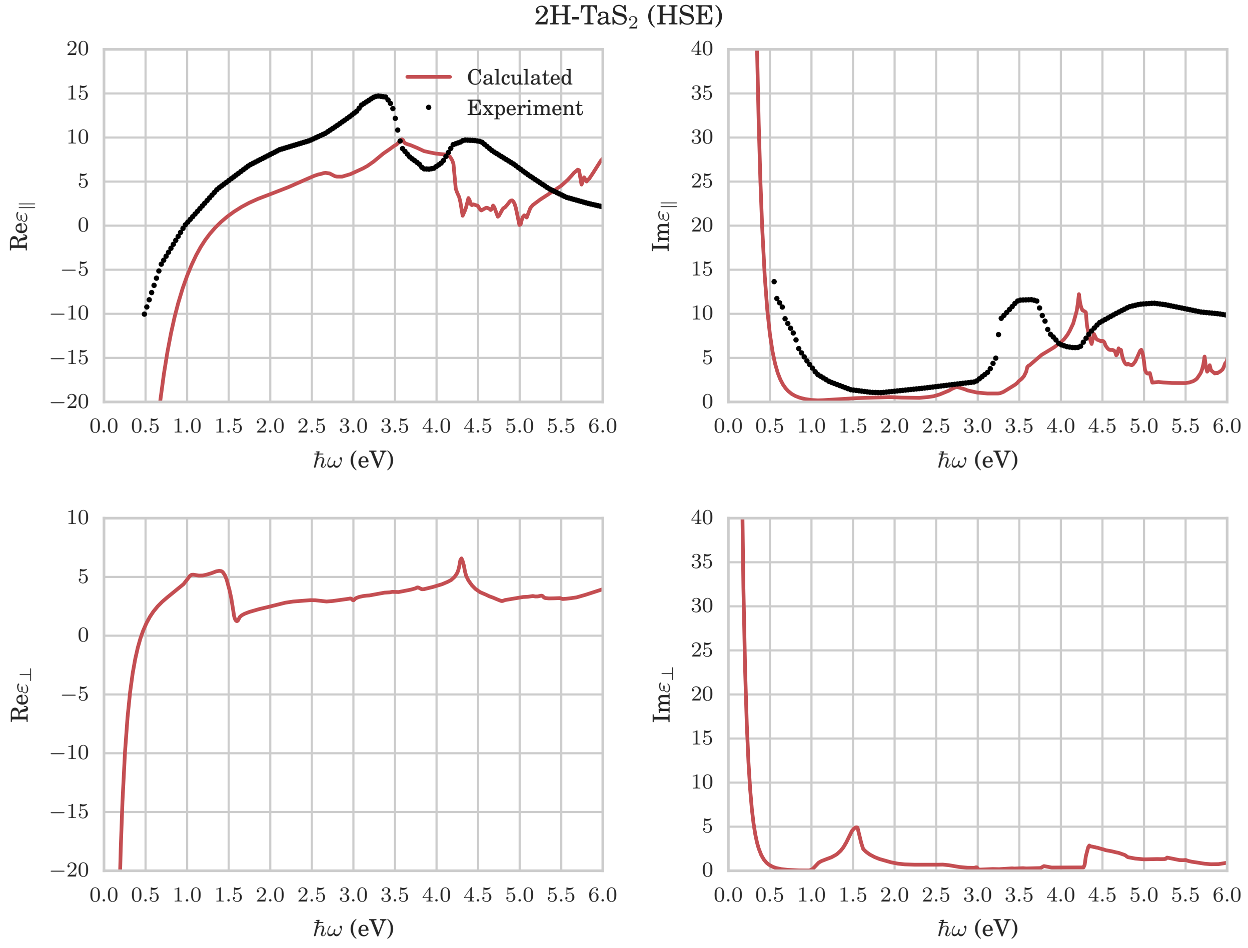

SUPPLEMENTARY FIGURE 14. **Calculated dielectric function of 2H-TaS$_2$** Calculated in-plane and out-of-plane components of the dielectric tensor of 2H-TaS$_2$ computed with HSE as explained in the Methods section. Experimental data from [4].

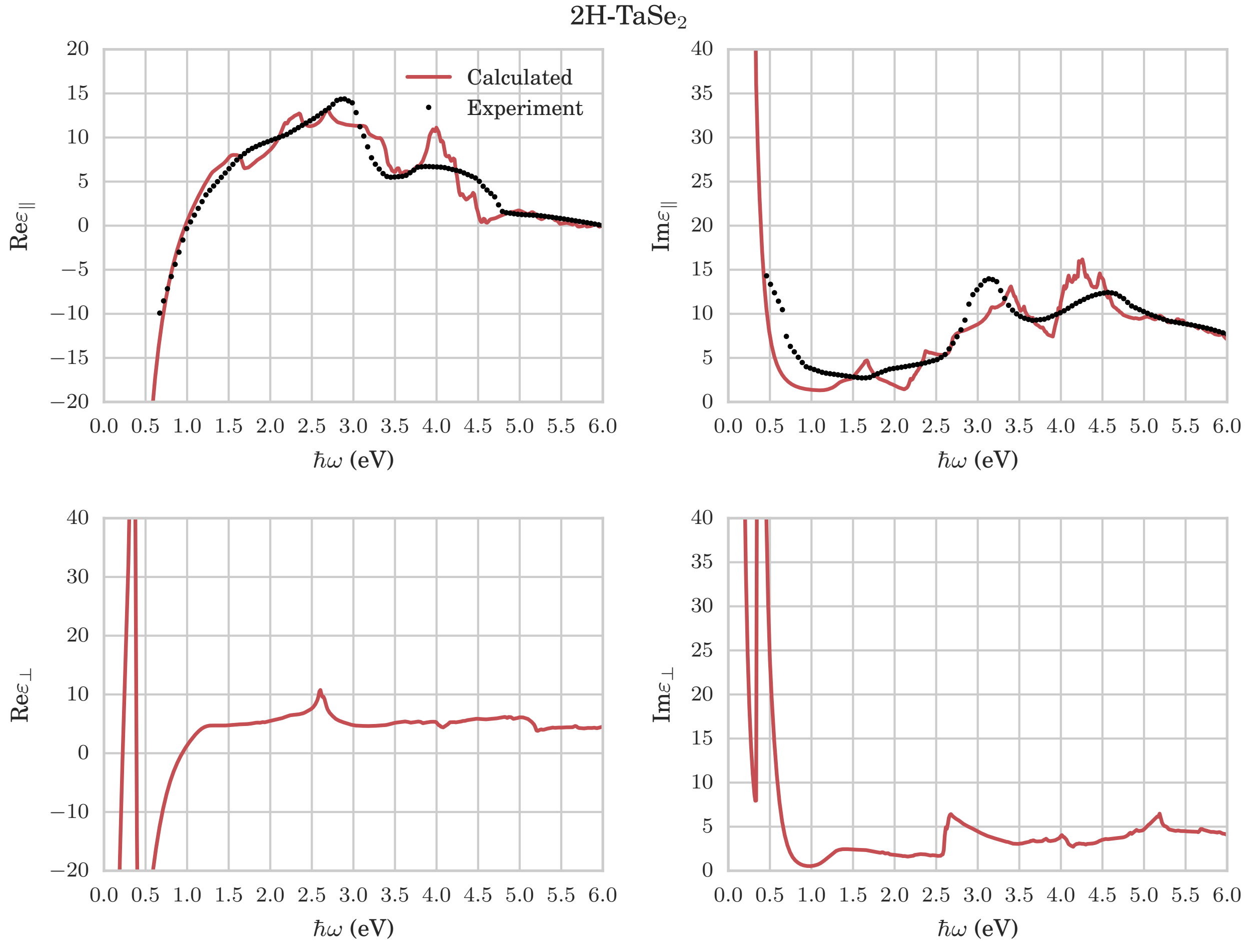

SUPPLEMENTARY FIGURE 15. **Calculated dielectric function of 2H-TaSe$_2$** Calculated in-plane and out-of-plane components of the dielectric tensor of 2H-TaSe$_2$. Experimental data from [4].

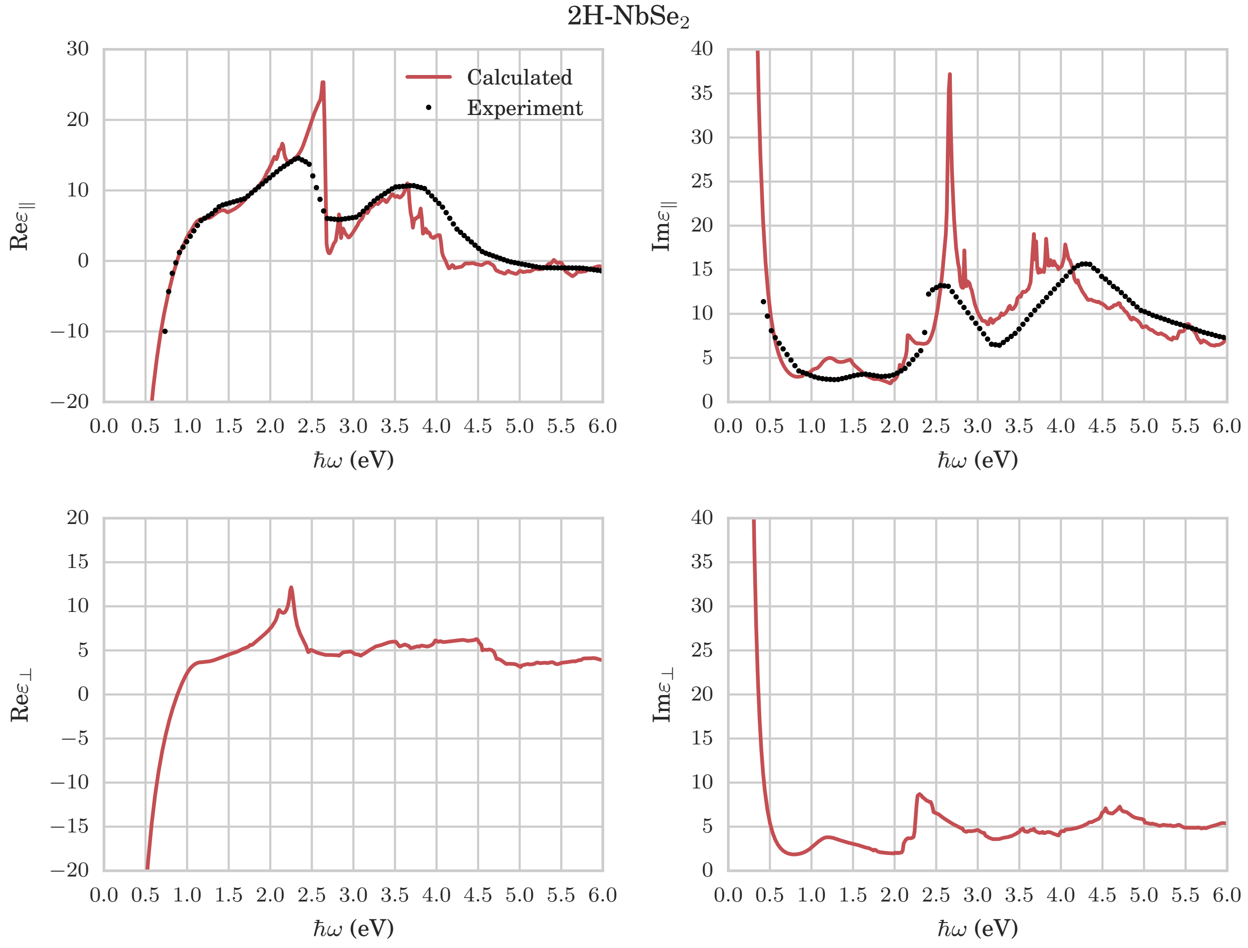

SUPPLEMENTARY FIGURE 16. **Calculated dielectric function of 2H-NbSe$_2$** Calculated in-plane and out-of-plane components of the dielectric tensor of 2H-NbSe$_2$. Experimental data from [4].

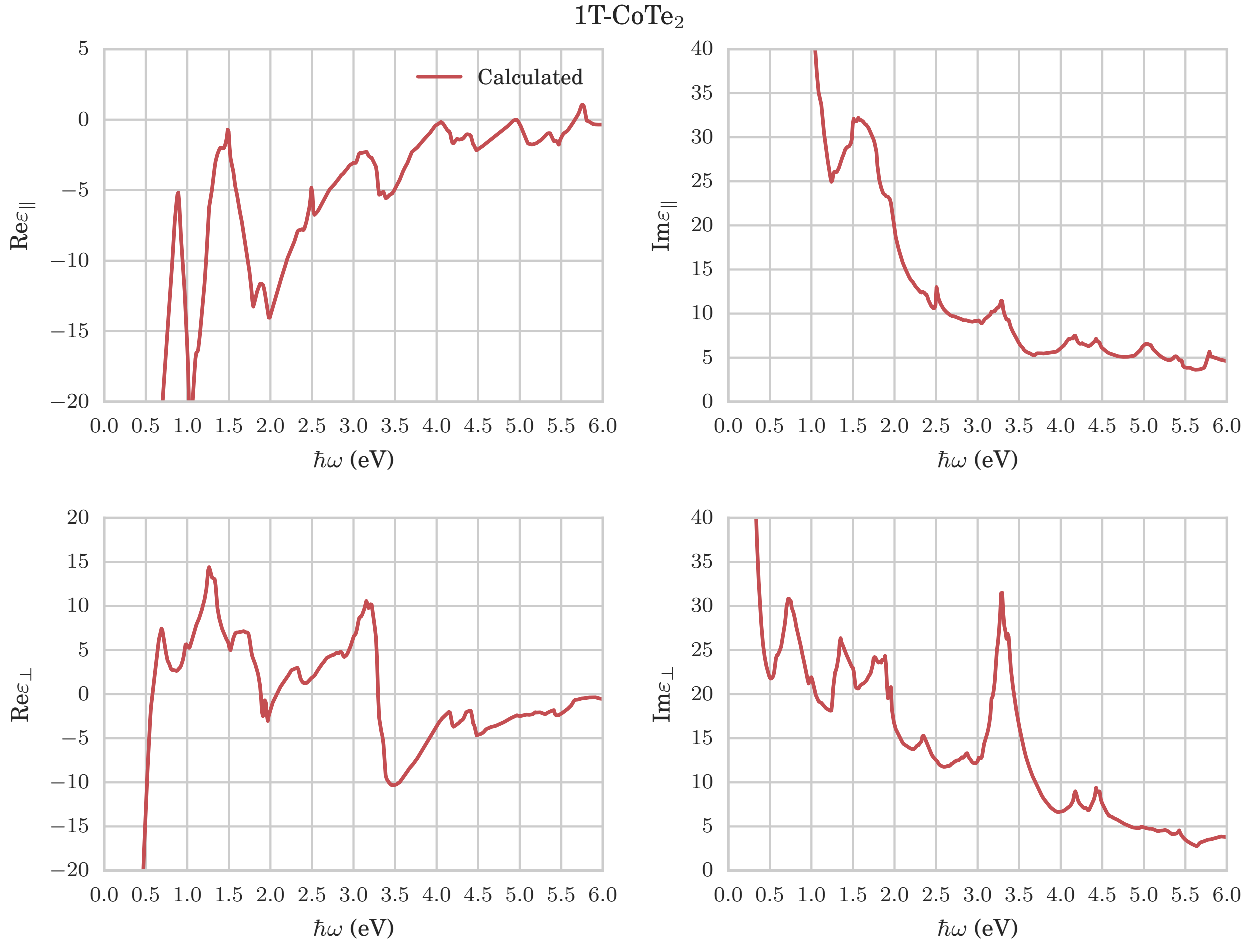

SUPPLEMENTARY FIGURE 17. **Calculated dielectric function of 1T-CoTe$_2$** Calculated in-plane and out-of-plane components of the dielectric tensor of 1T-CoTe$_2$.

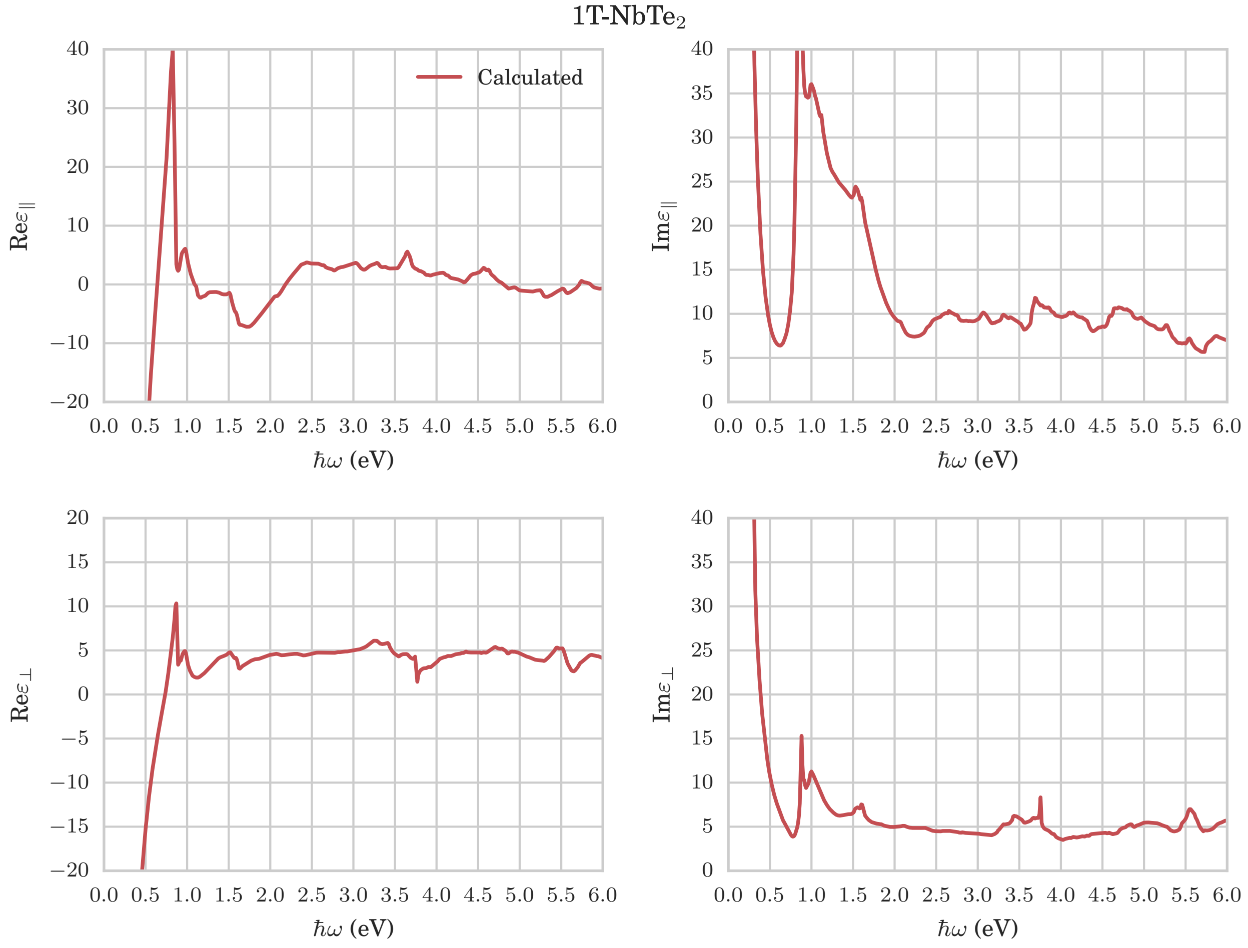

SUPPLEMENTARY FIGURE 18. **Calculated dielectric function of 1T-NbTe$_2$** Calculated in-plane and out-of-plane components of the dielectric tensor of 1T-NbTe$_2$.

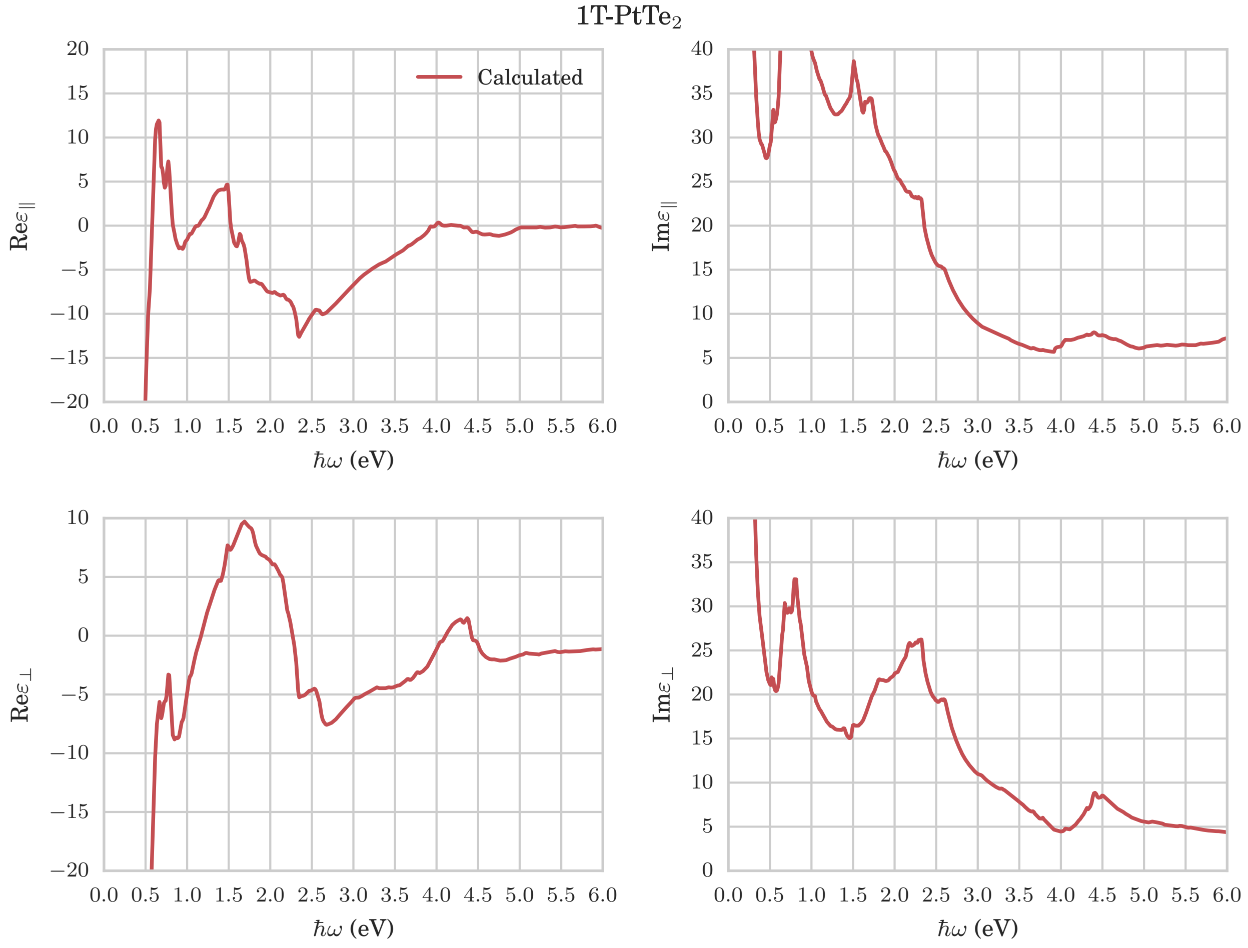

SUPPLEMENTARY FIGURE 19. **Calculated dielectric function of 1T-PtTe$_2$** Calculated in-plane and out-of-plane components of the dielectric tensor of 1T-PtTe$_2$.

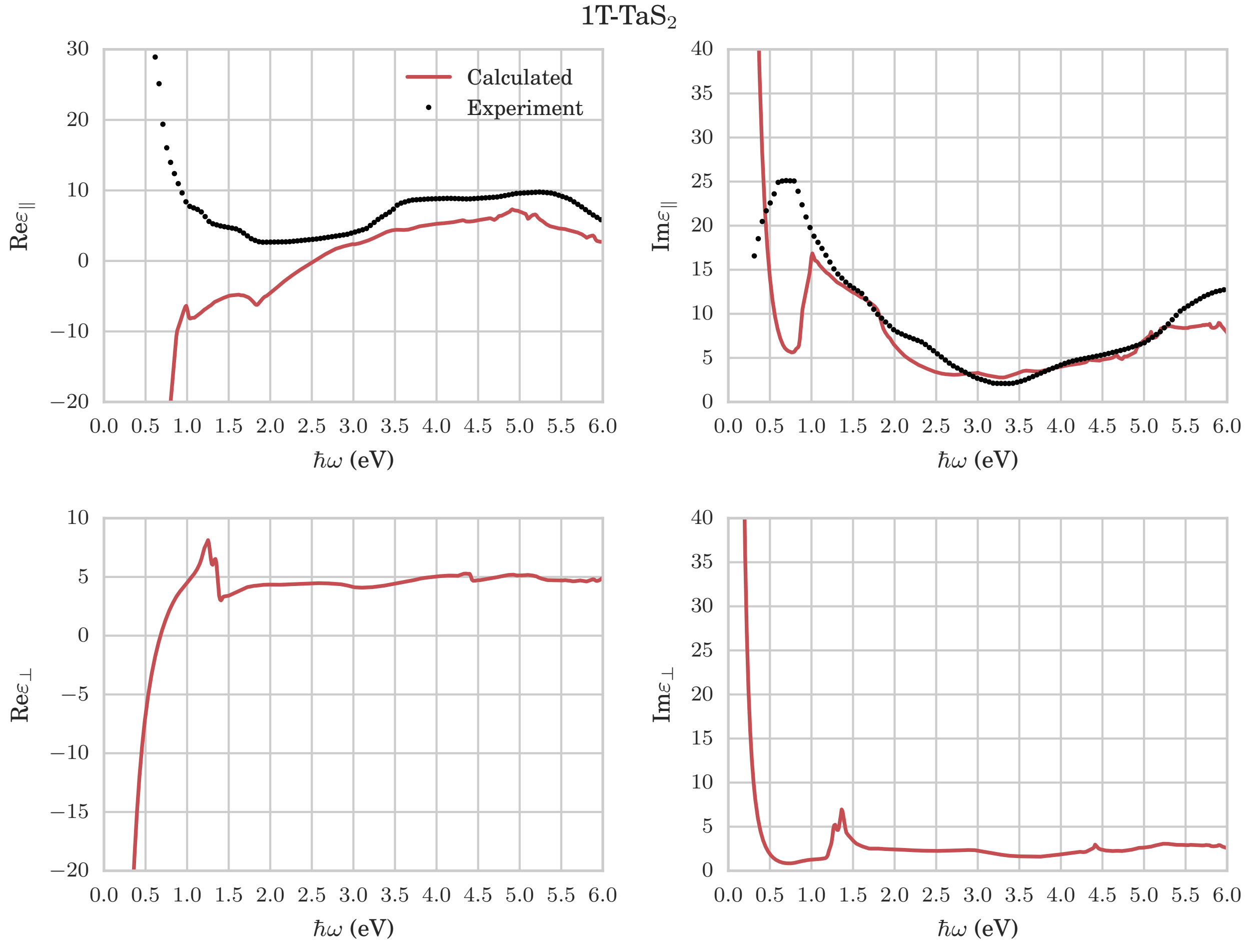

SUPPLEMENTARY FIGURE 20. **Calculated dielectric function of 1T-TaS$_2$** Calculated in-plane and out-of-plane components of the dielectric tensor of 1T-TaS$_2$. Experimental data from [4]. This large discrepancy is probably due to the charge density wave of 1T-TaS$_2$.

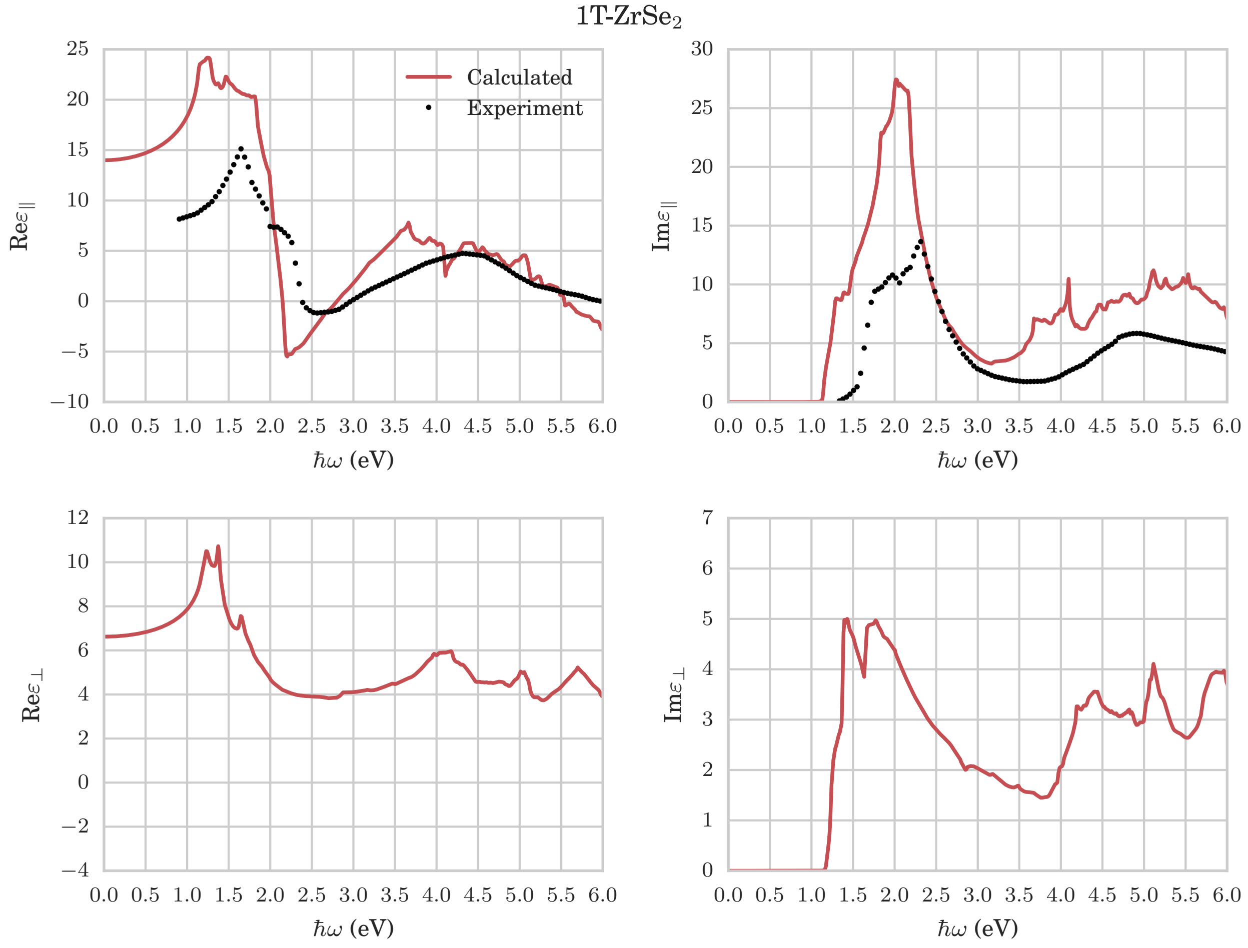

SUPPLEMENTARY FIGURE 21. **Calculated dielectric function of 1T-ZrSe$_2$** Calculated in-plane and out-of-plane components of the dielectric tensor of 1T-ZrSe$_2$. Experimental data from [9].

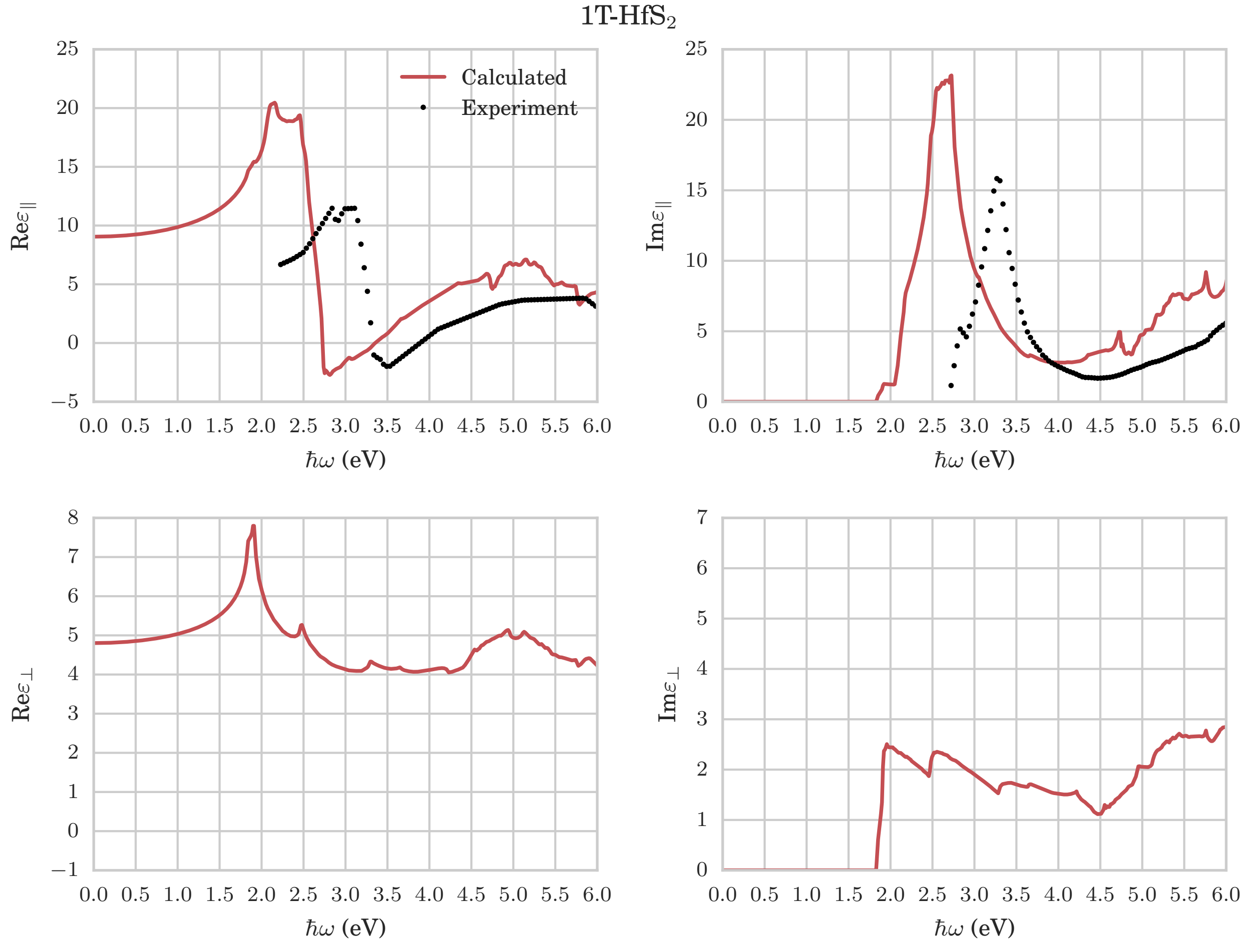

SUPPLEMENTARY FIGURE 22. **Calculated dielectric function of 1T-HfS$_2$** Calculated in-plane and out-of-plane components of the dielectric tensor of 1T-HfS$_2$. Experimental data from [9].

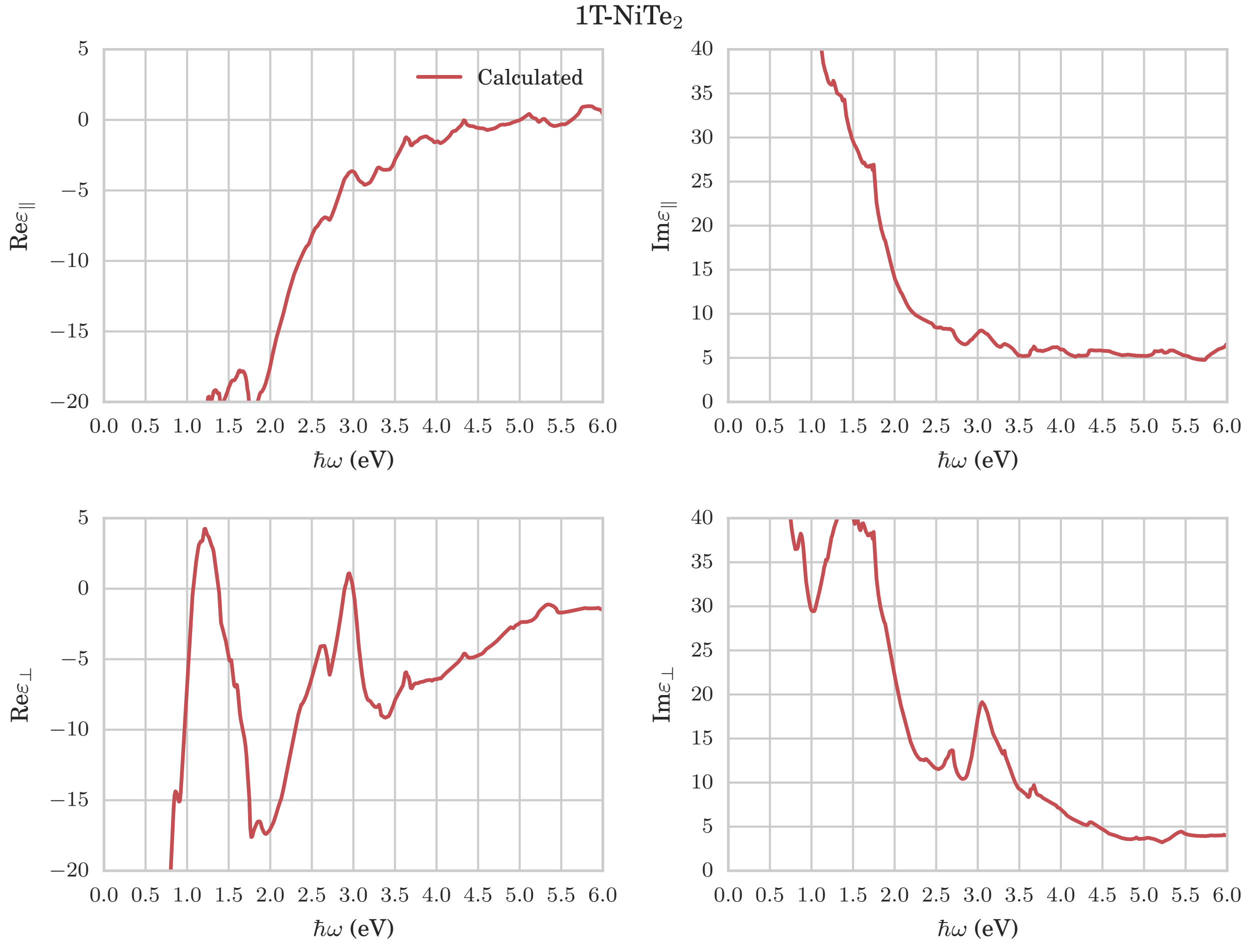

SUPPLEMENTARY FIGURE 23. **Calculated dielectric function of 1T-NiTe$_2$** Calculated in-plane and out-of-plane components of the dielectric tensor of 1T-NiTe$_2$.

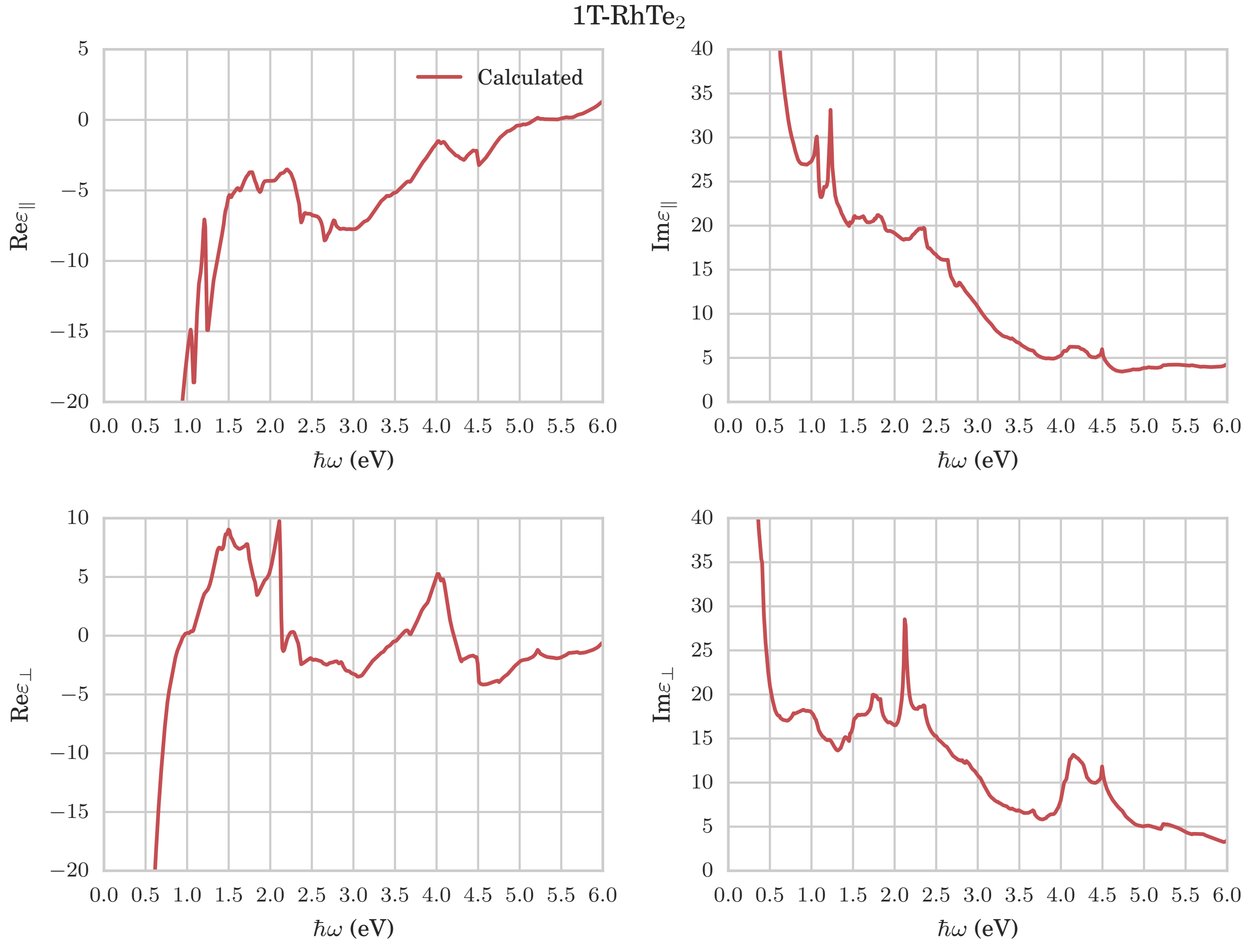

SUPPLEMENTARY FIGURE 24. **Calculated dielectric function of 1T-RhTe$_2$** Calculated in-plane and out-of-plane components of the dielectric tensor of 1T-RhTe$_2$.

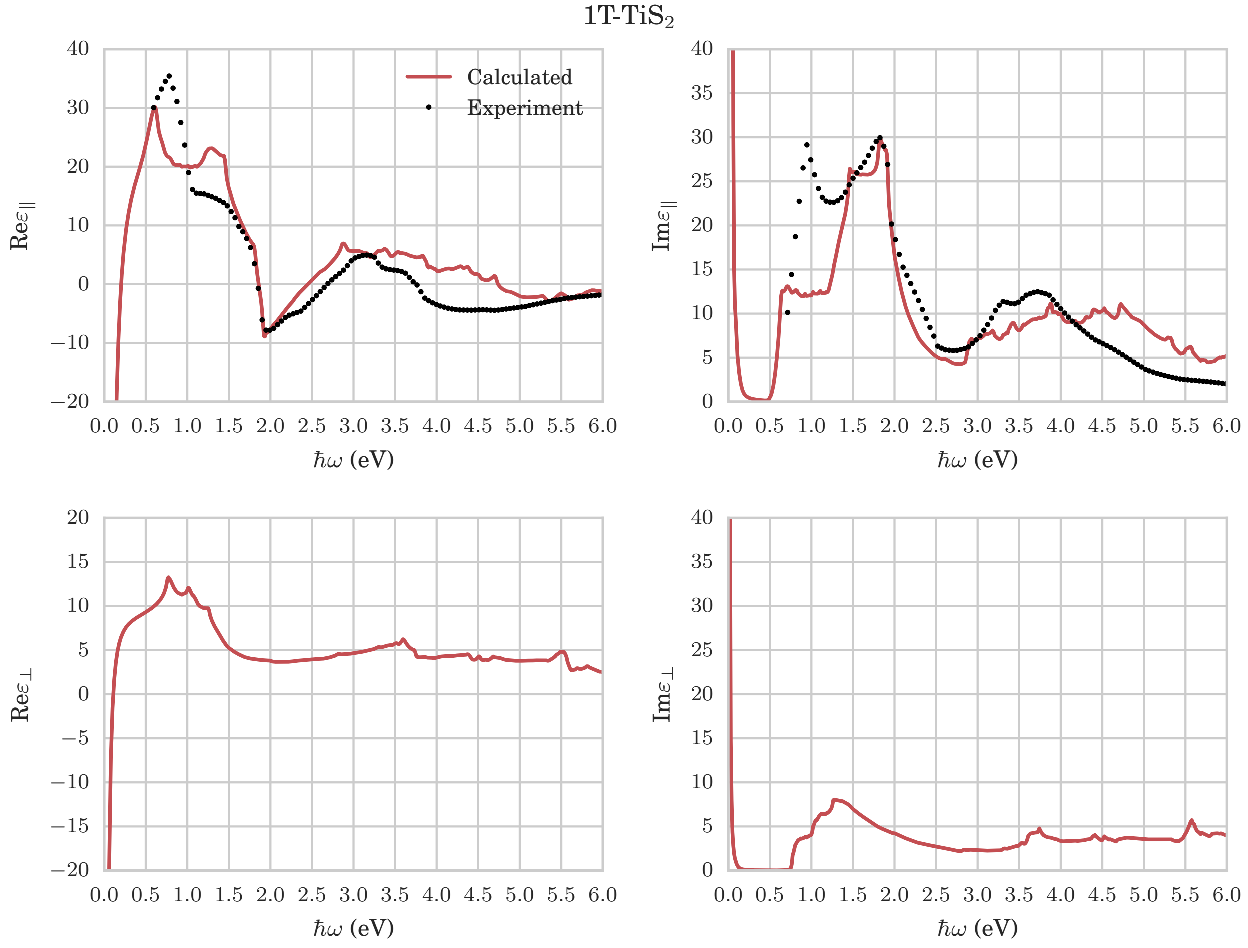

SUPPLEMENTARY FIGURE 25. **Calculated dielectric function of 1T-TiS$_2$** Calculated in-plane and out-of-plane components of the dielectric tensor of 1T-TiS$_2$. Experimental data from [9].

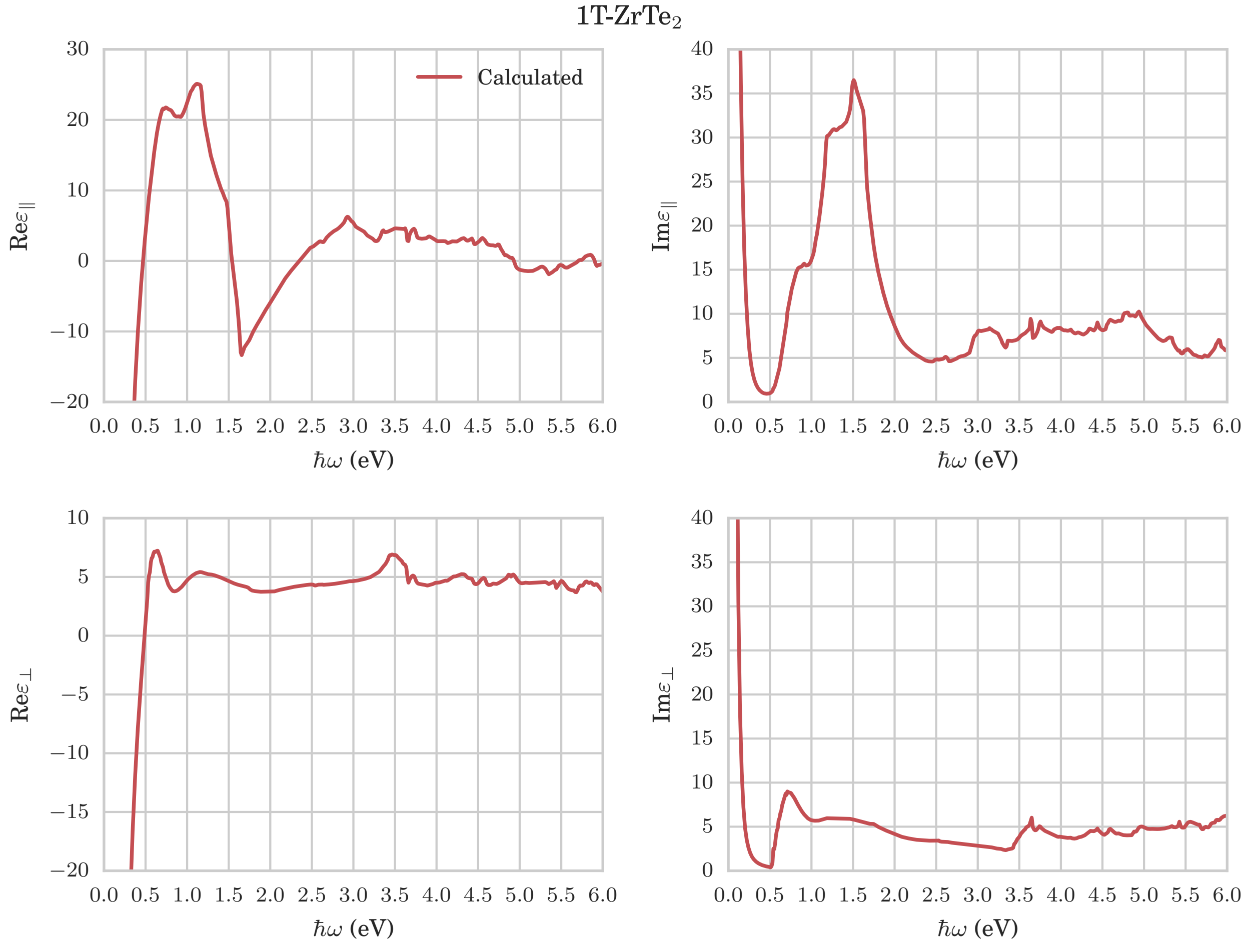

SUPPLEMENTARY FIGURE 26. **Calculated dielectric function of 1T-ZrTe$_2$** Calculated in-plane and out-of-plane components of the dielectric tensor of 1T-ZrTe$_2$.

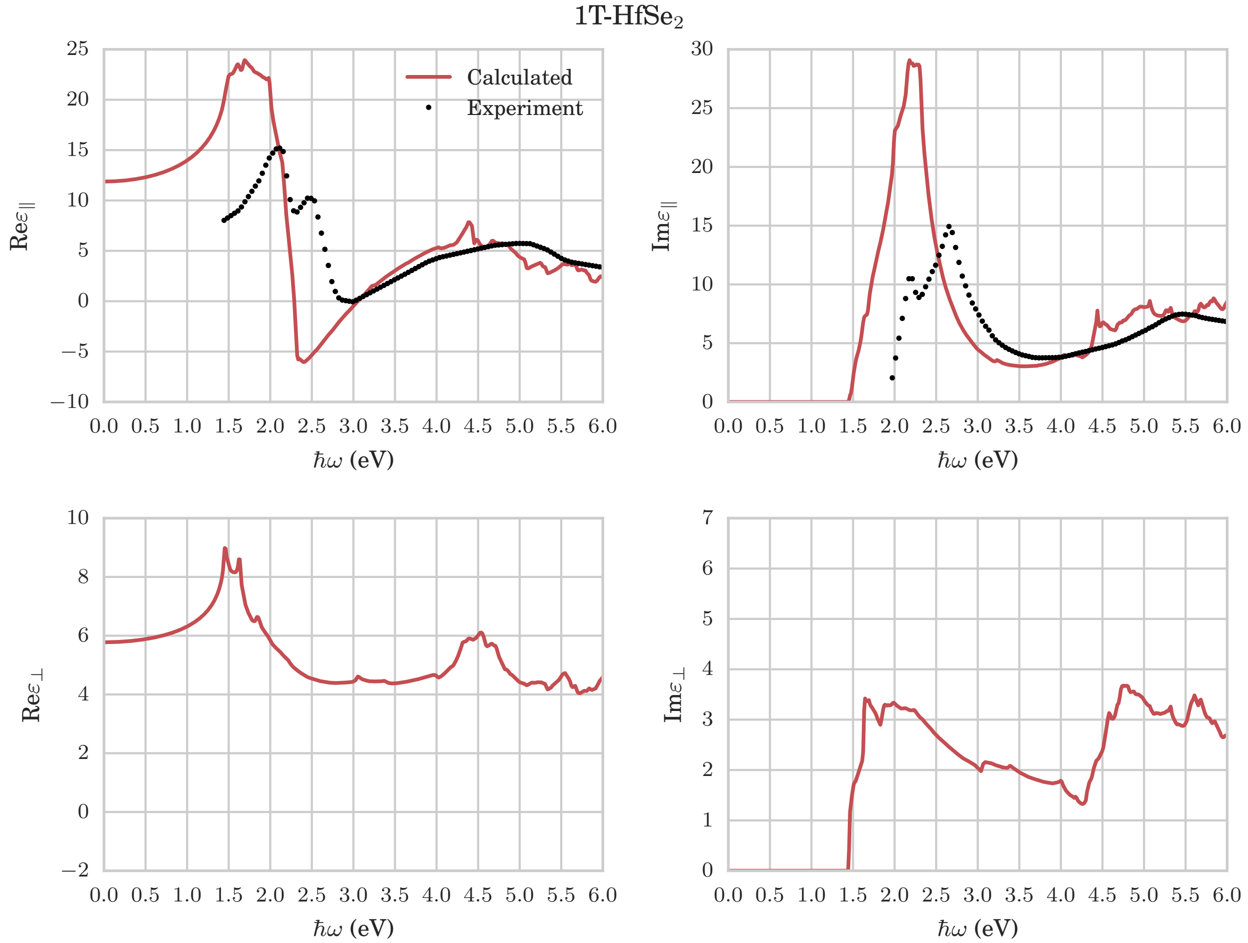

SUPPLEMENTARY FIGURE 27. **Calculated dielectric function of 1T-HfSe$_2$** Calculated in-plane and out-of-plane components of the dielectric tensor of 1T-HfSe$_2$. Experimental data from [9].

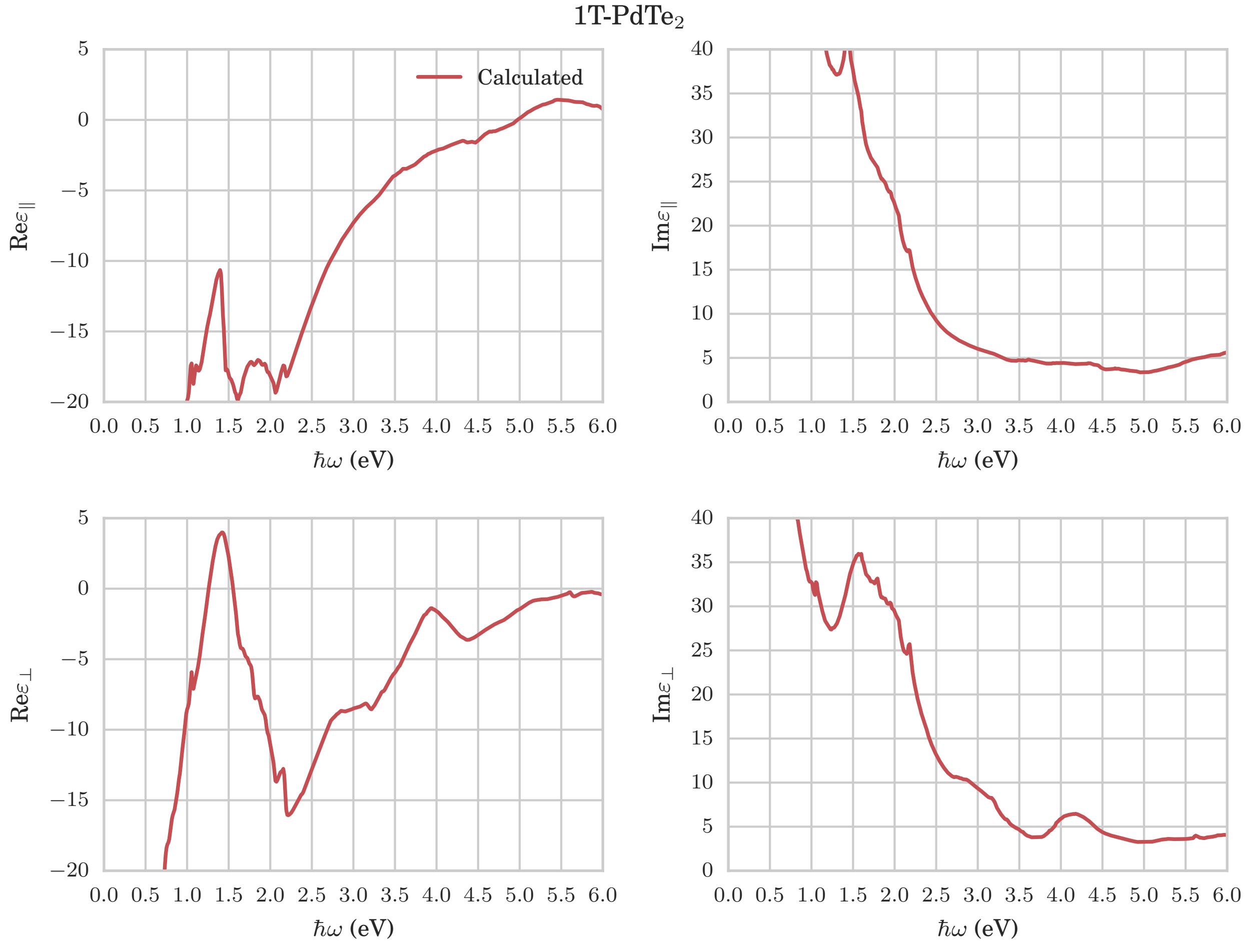

SUPPLEMENTARY FIGURE 28. **Calculated dielectric function of 1T-PdTe$_2$** Calculated in-plane and out-of-plane components of the dielectric tensor of 1T-PdTe$_2$.

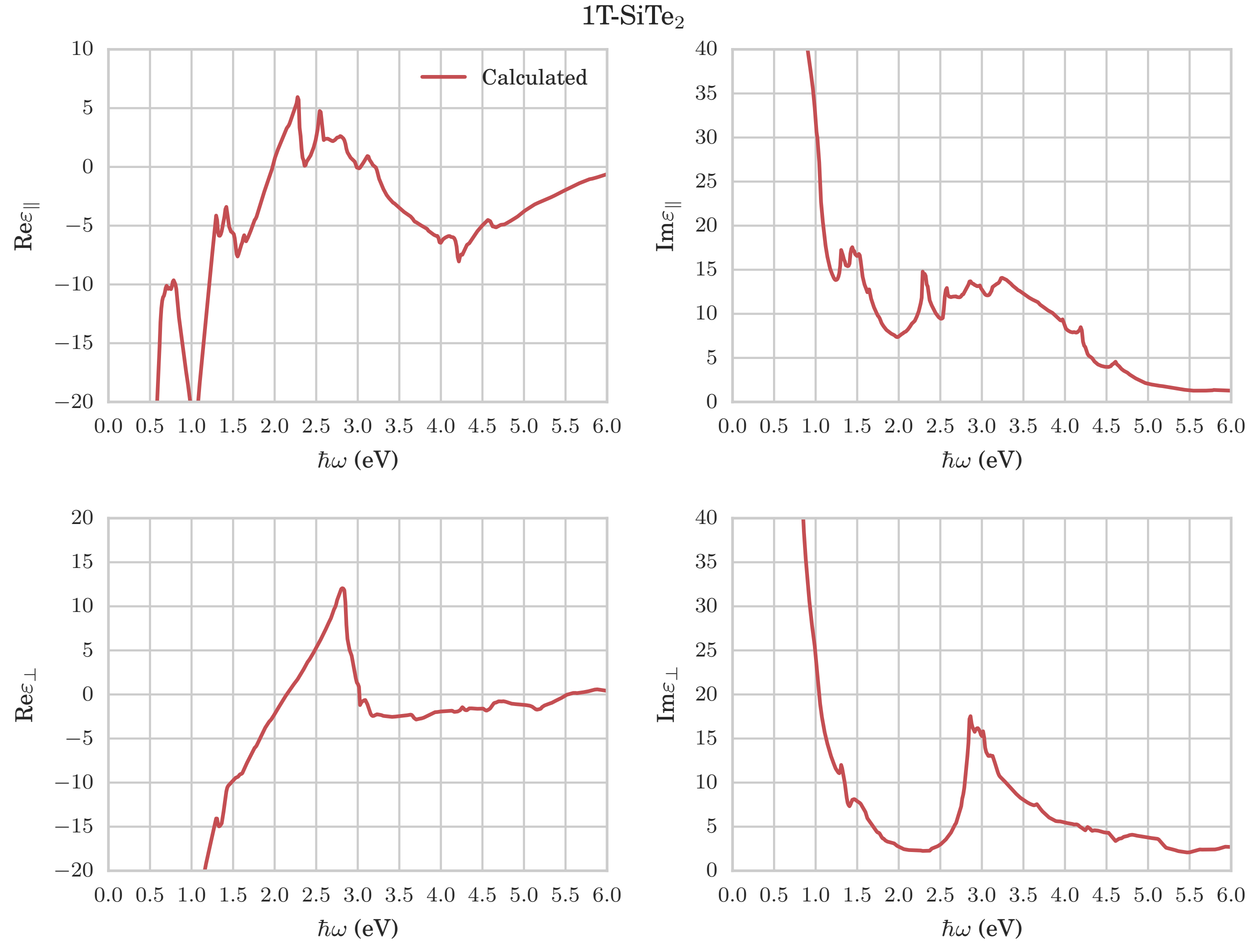

SUPPLEMENTARY FIGURE 29. **Calculated dielectric function of 1T-SiTe$_2$** Calculated in-plane and out-of-plane components of the dielectric tensor of 1T-SiTe$_2$.

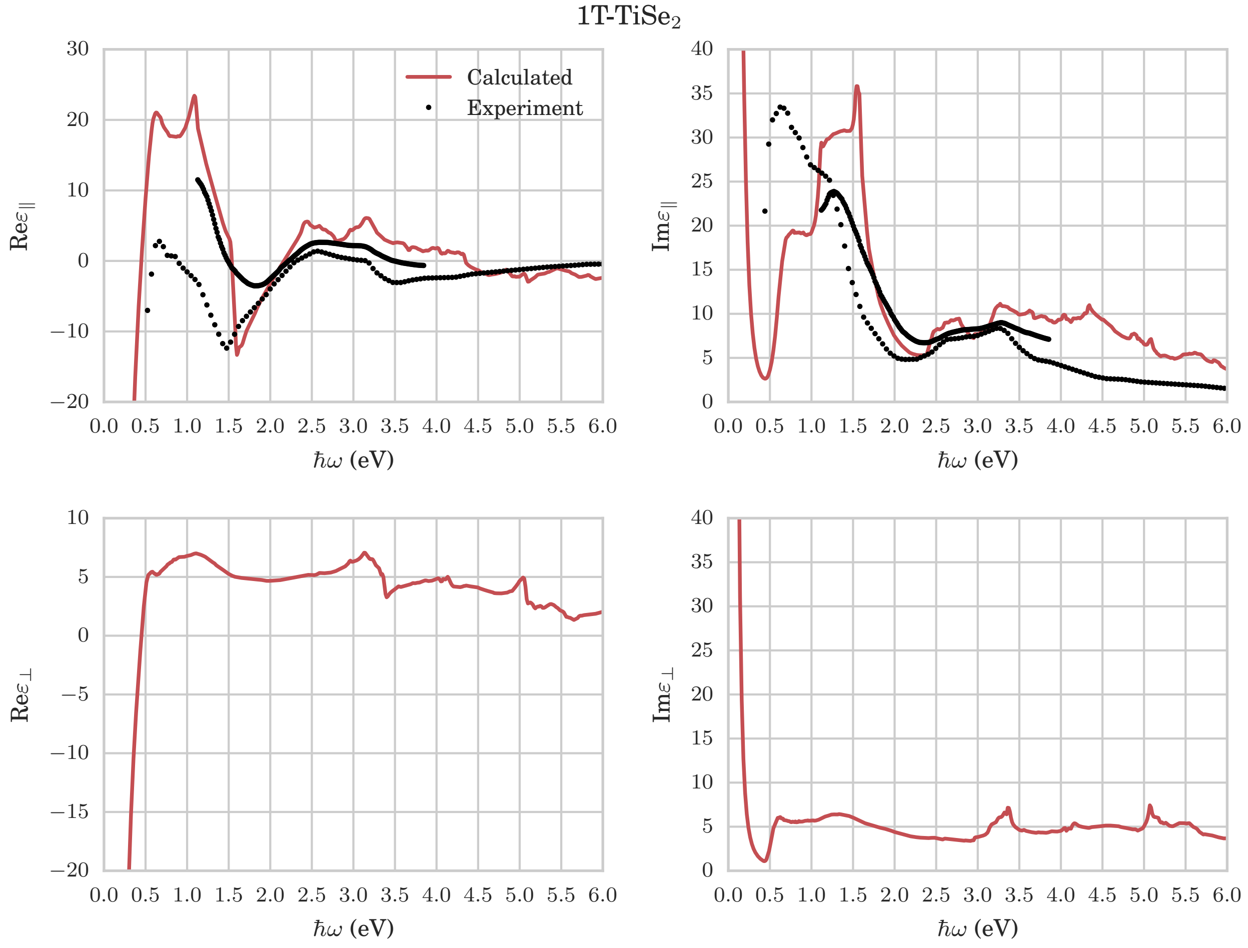

SUPPLEMENTARY FIGURE 30. **Calculated dielectric function of 1T-TiSe$_2$** Calculated in-plane and out-of-plane components of the dielectric tensor of 1T-TiSe$_2$. Experimental data from [9, 10].

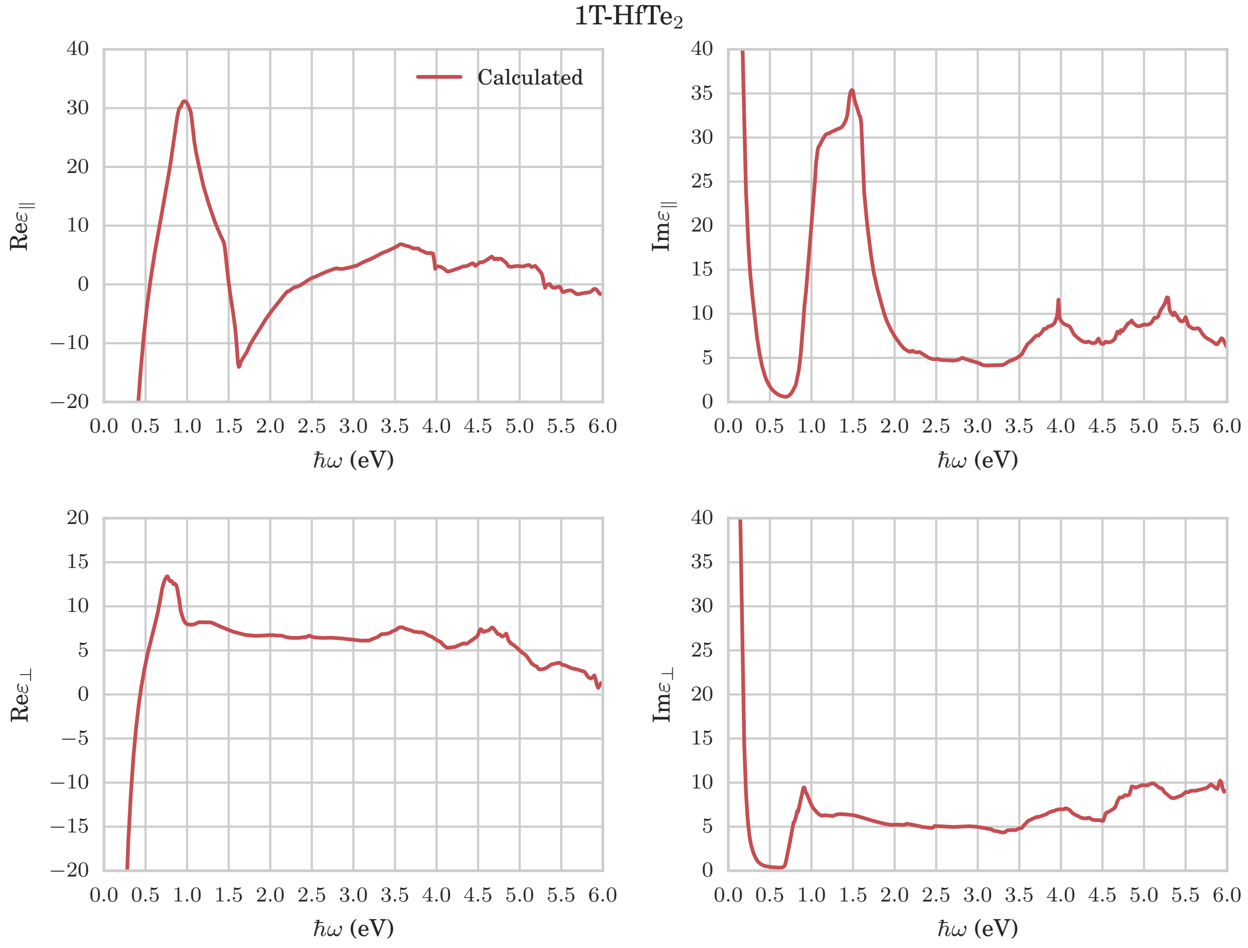

SUPPLEMENTARY FIGURE 31. **Calculated dielectric function of 1T-HfTe$_2$** Calculated in-plane and out-of-plane components of the dielectric tensor of 1T-HfTe$_2$.

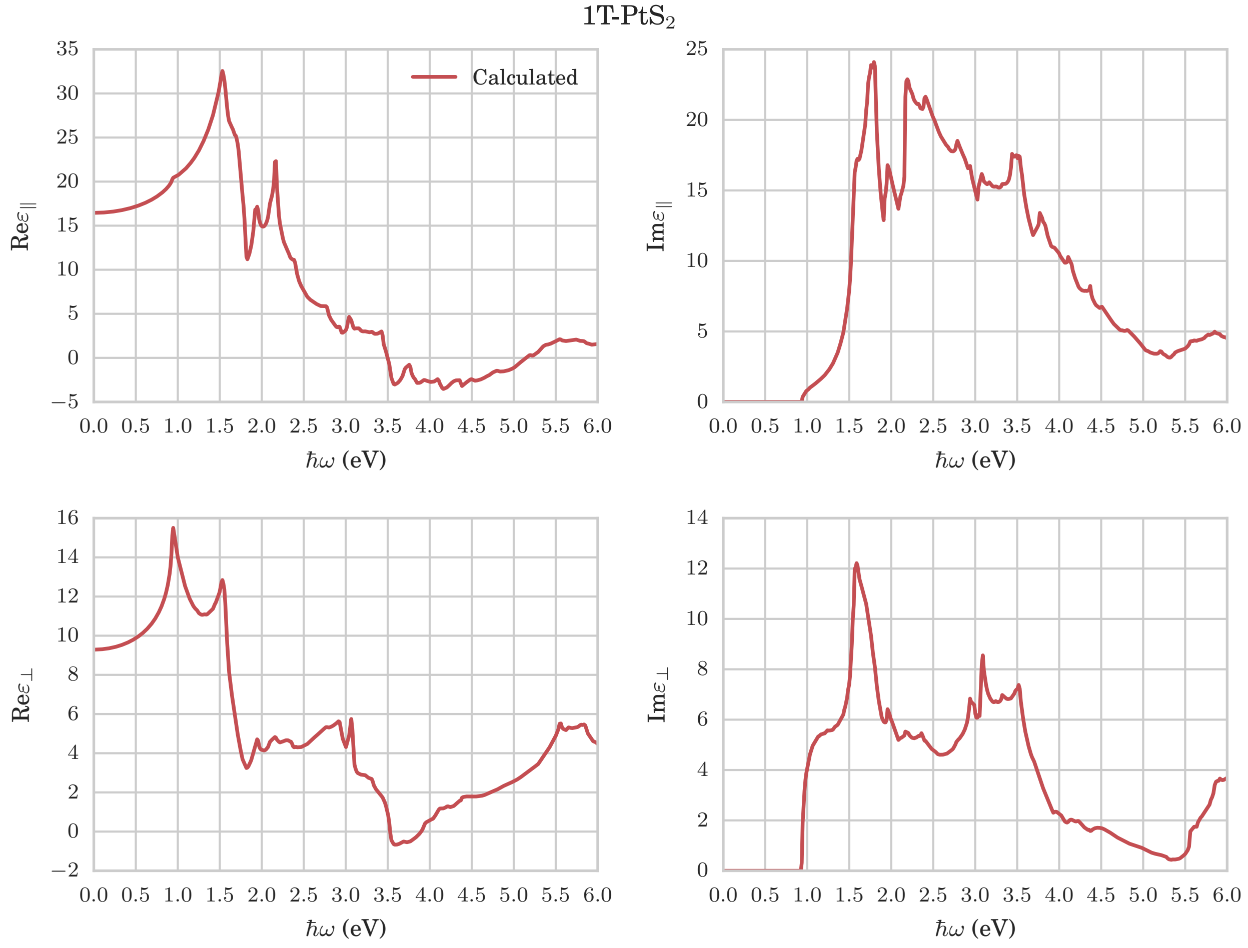

SUPPLEMENTARY FIGURE 32. **Calculated dielectric function of 1T-PtS$_2$** Calculated in-plane and out-of-plane components of the dielectric tensor of 1T-PtS$_2$.

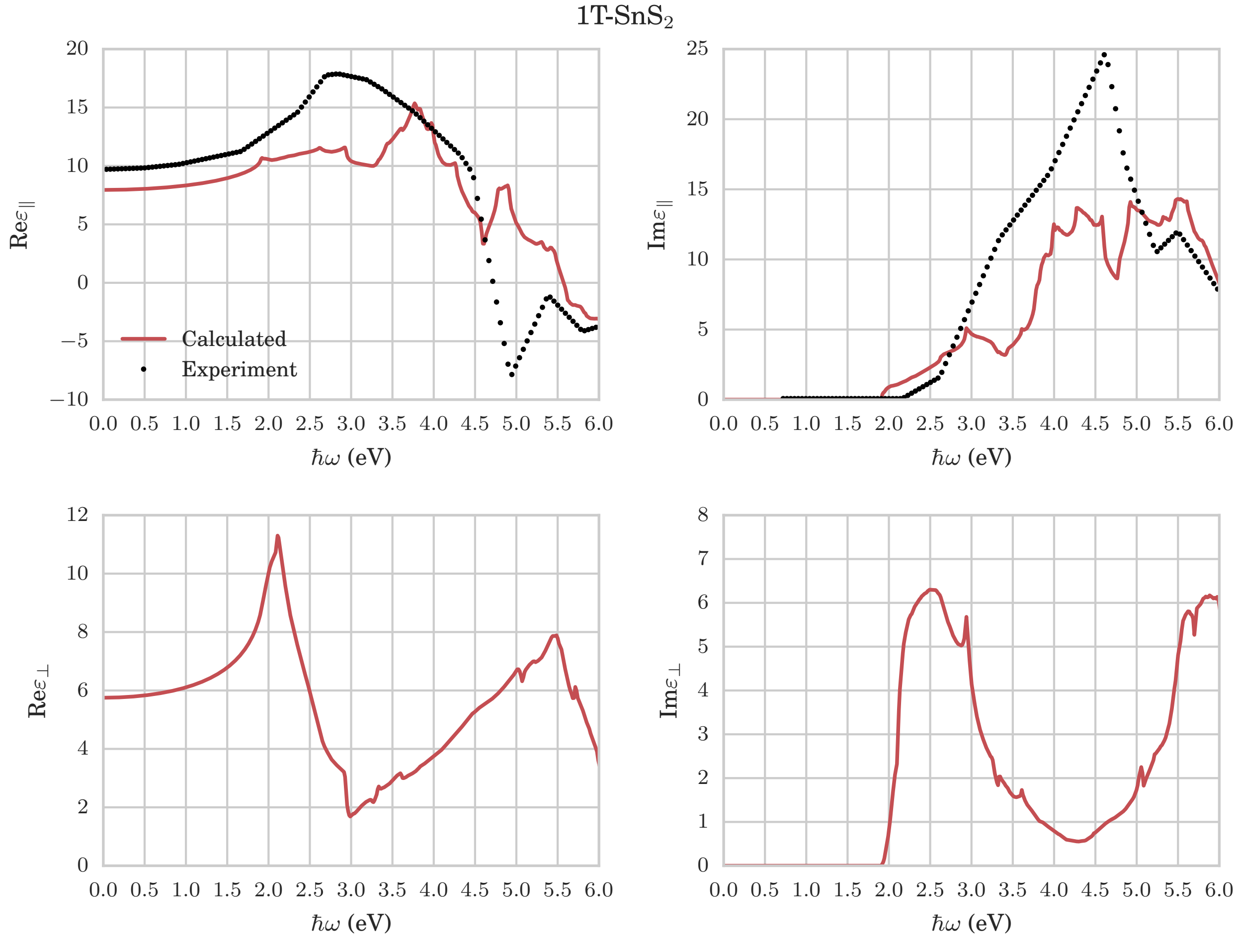

SUPPLEMENTARY FIGURE 33. **Calculated dielectric function of 1T-SnS$_2$** Calculated in-plane and out-of-plane components of the dielectric tensor of 1T-SnS$_2$. Experimental data from [11].

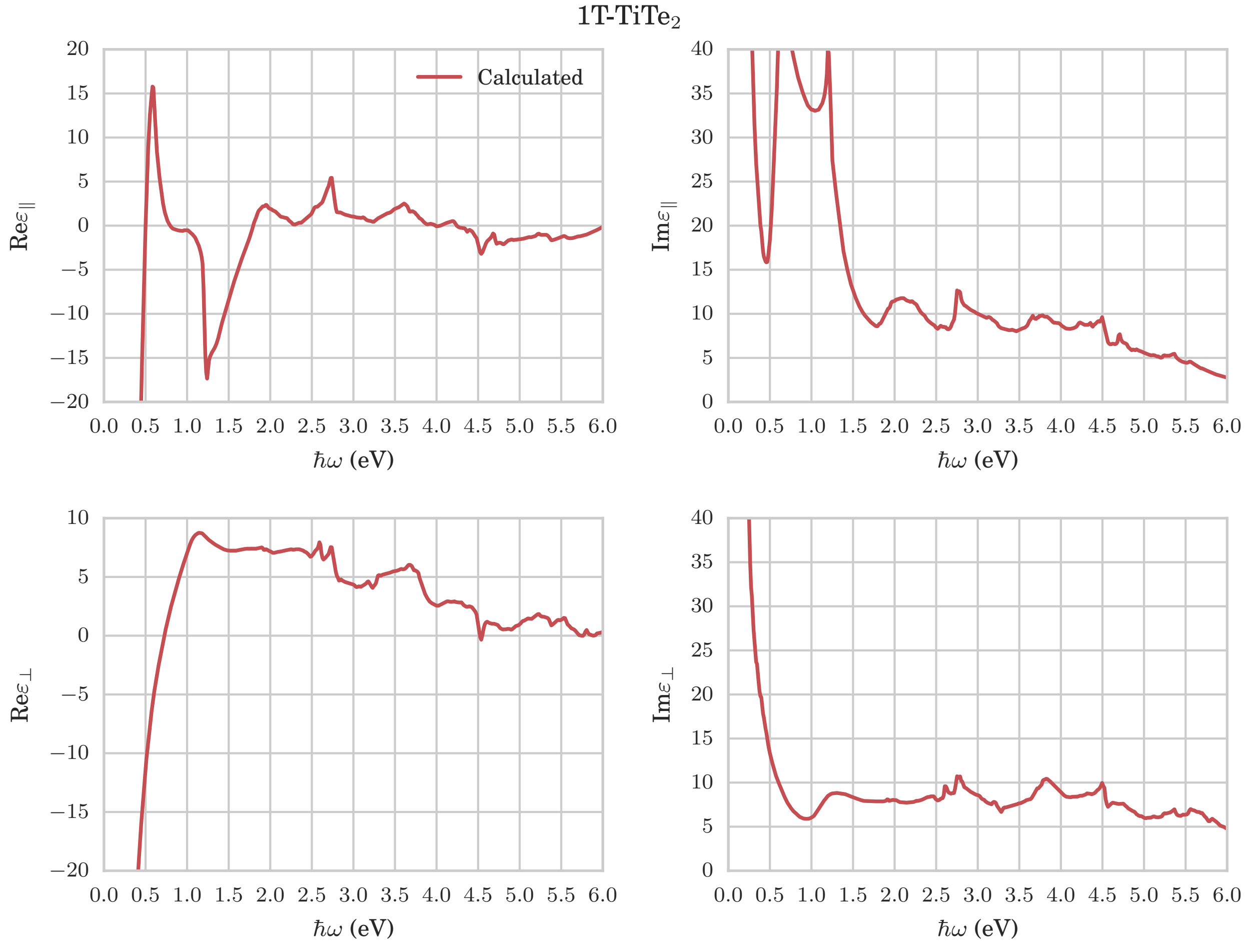

SUPPLEMENTARY FIGURE 34. **Calculated dielectric function of 1T-$TiTe_2$** Calculated in-plane and out-of-plane components of the dielectric tensor of 1T-$TiTe_2$.

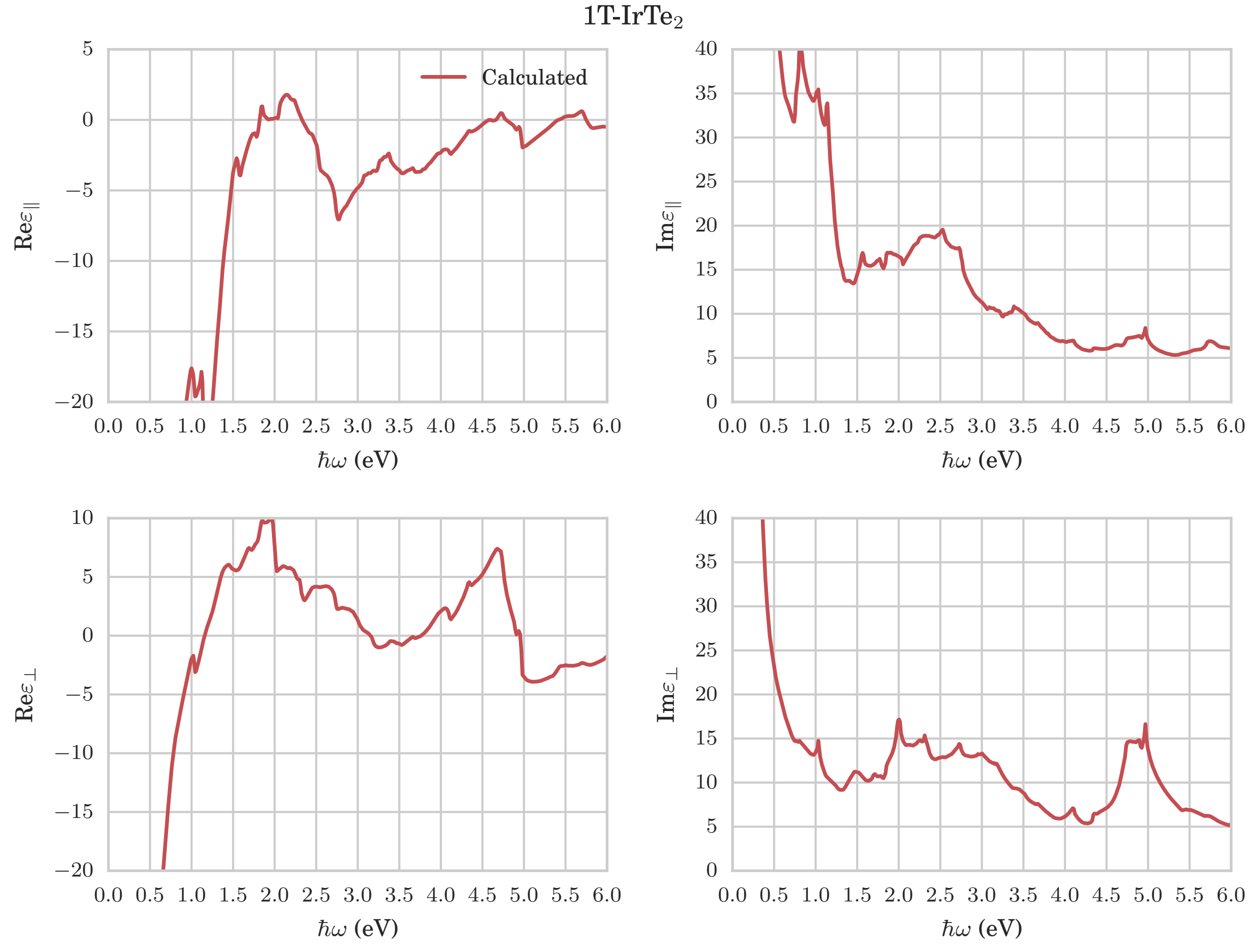

SUPPLEMENTARY FIGURE 35. **Calculated dielectric function of 1T-IrTe$_2$** Calculated in-plane and out-of-plane components of the dielectric tensor of 1T-IrTe$_2$.

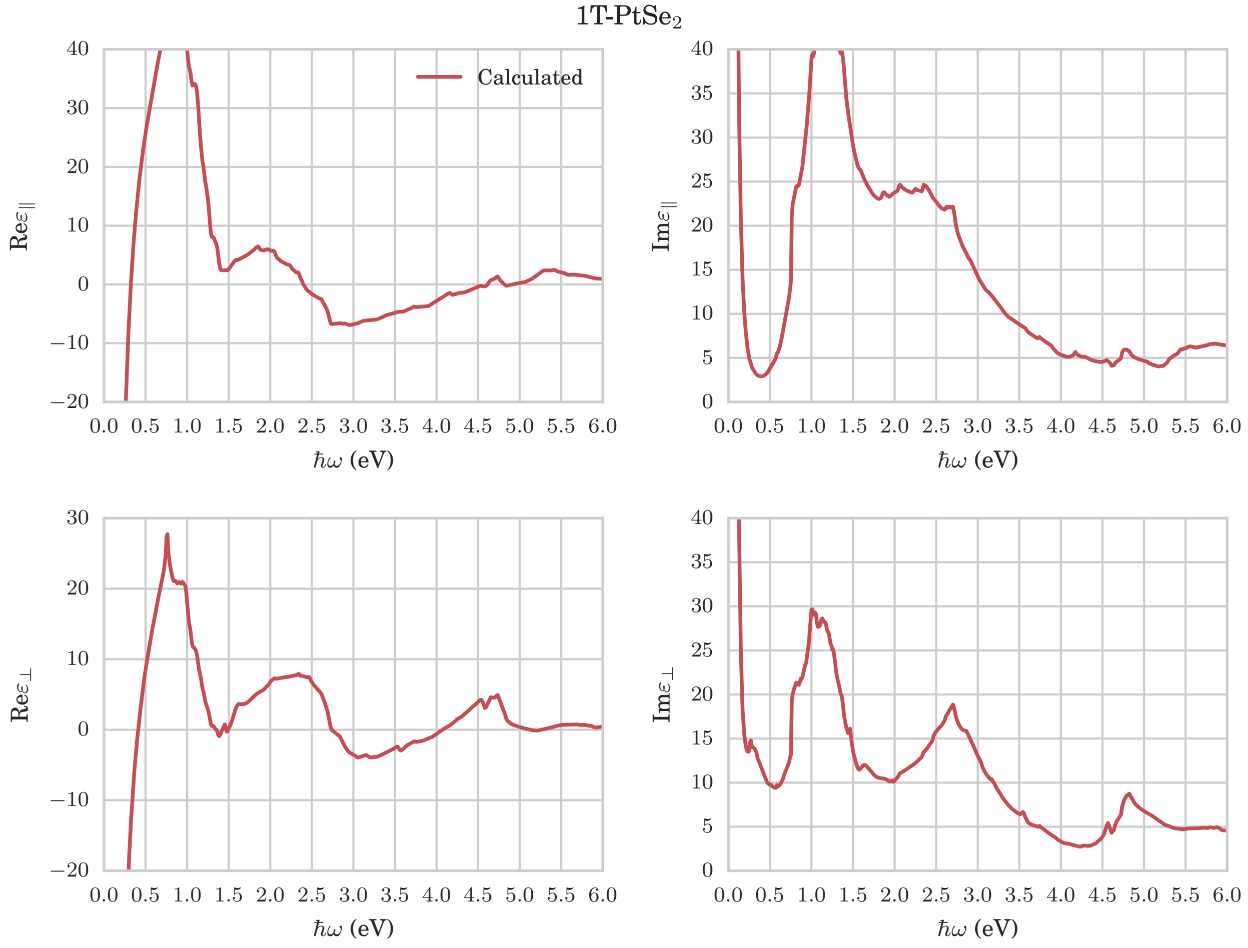

SUPPLEMENTARY FIGURE 36. **Calculated dielectric function of 1T-PtSe$_2$** Calculated in-plane and out-of-plane components of the dielectric tensor of 1T-PtSe$_2$.

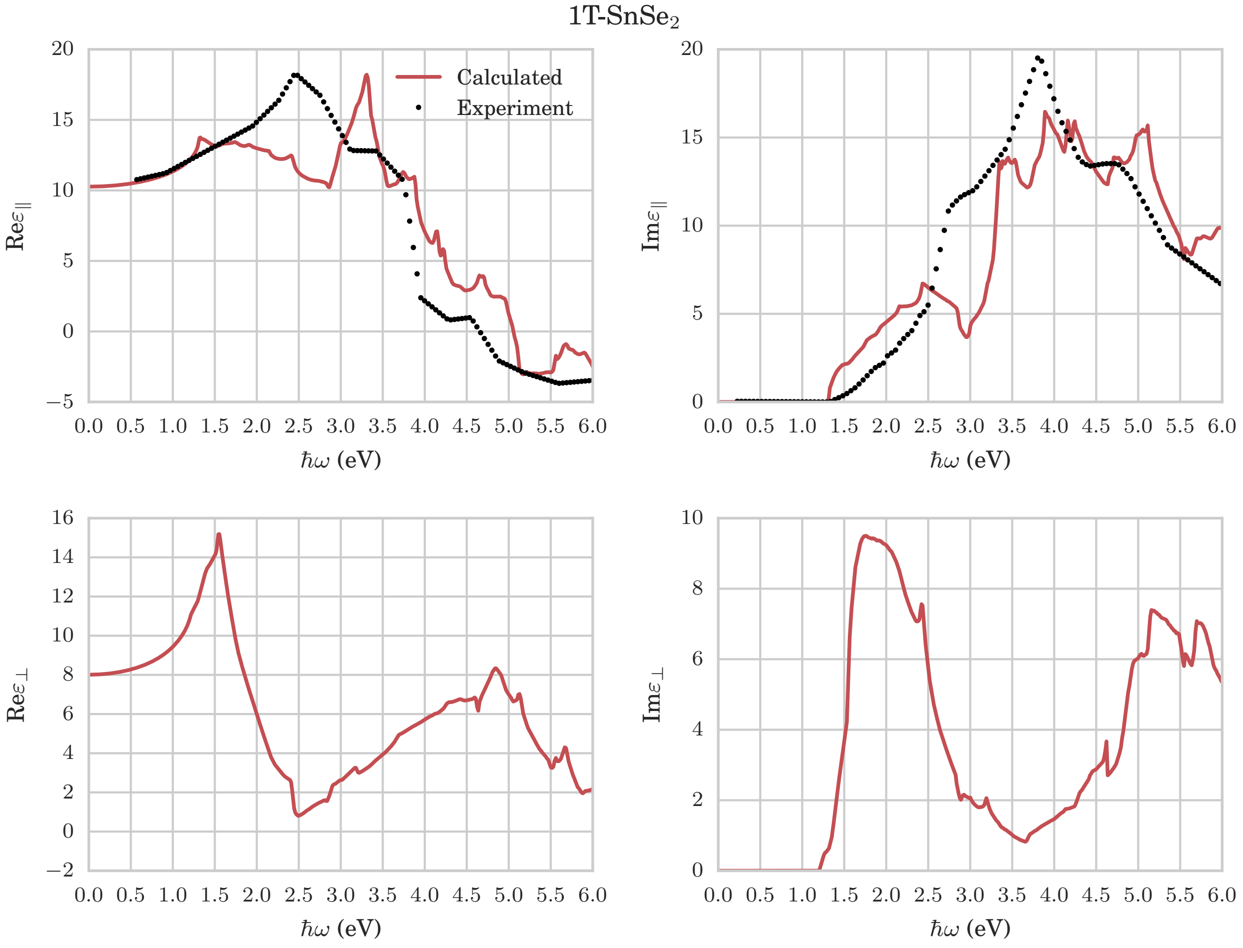

SUPPLEMENTARY FIGURE 37. **Calculated dielectric function of 1T-SnSe$_2$** Calculated in-plane and out-of-plane components of the dielectric tensor of 1T-SnSe$_2$. Experimental data from [11].

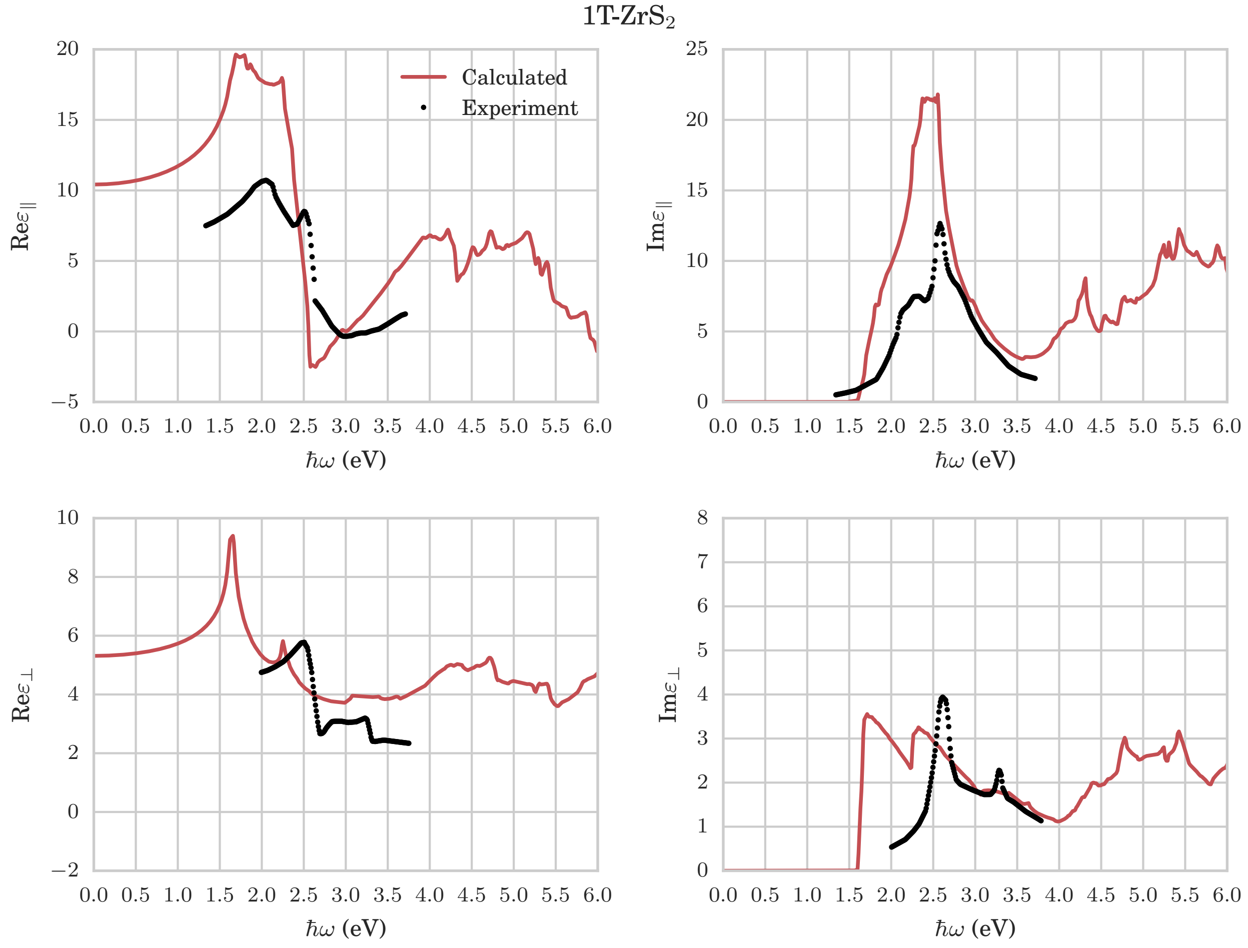

SUPPLEMENTARY FIGURE 38. **Calculated dielectric function of 1T-ZrS$_2$** Calculated in-plane and out-of-plane components of the dielectric tensor of 1T-ZrS$_2$. Experimental data from [8].

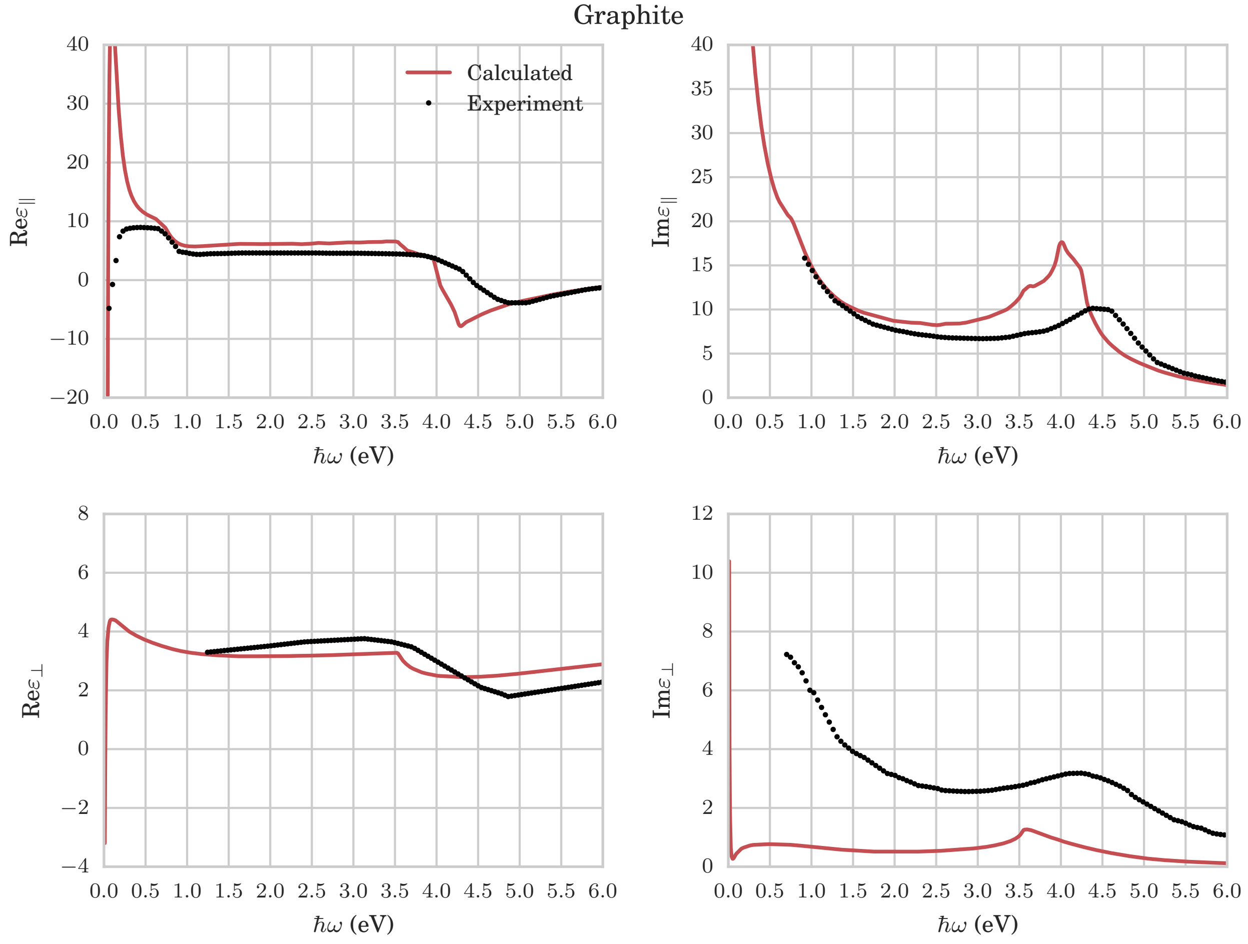

SUPPLEMENTARY FIGURE 39. **Calculated dielectric function of Graphite** Calculated in-plane and out-of-plane components of the dielectric tensor of Graphite. Experimental data from [5].

SUPPLEMENTARY REFERENCES

[1] Yan, W., Wubs, M. & Mortensen, N. A. Hyperbolic metamaterials: Nonlocal response regularizes broadband supersingularity. *Physical Review B* **86**, 205429 (2012).
[2] Christensen, T. *et al.* Nonlocal Response of Metallic Nanospheres Probed by Light, Electrons, and Atoms. *ACS Nano* **8**, 1745–1758 (2014).
[3] Wang, W., Xiao, S. & Mortensen, N. A. Localized plasmons in bilayer graphene nanodisks. *Phys. Rev. B* **93**, 165407 (2016).
[4] Beal, A. R., Hughes, H. P. & Liang, W. Y. The reflectivity spectra of some group VA transition metal dichalcogenides. *Journal of Physics C: Solid State Physics* **8**, 4236 (1975).
[5] Taft, E. A. & Philipp, H. R. Optical Properties of Graphite. *Physical Review* **138**, A197—-A202 (1965).
[6] Beal, A. R. & Hughes, H. P. Kramers-Kronig analysis of the reflectivity spectra of 2H-MoS_2 , 2H-$MoSe_2$ and 2H-$MoTe_2$. *Journal of Physics C: Solid State Physics* **12**, 881 (1979).
[7] Zeppenfeld, K. Electron energy losses and optical anisotropy of MoS_2 single crystals. *Optics Communications* **1**, 377–378 (1970).
[8] Bayliss, S. C. & Liang, W. Y. Symmetry dependence of optical transitions in group 4B transition metal dichalcogenides. *Journal of Physics C: Solid State Physics* **15**, 1283 (1982).
[9] Bayliss, S. C. & Liang, W. Y. Reflectivity, joint density of states and band structure of group IVb transition-metal dichalcogenides. *Journal of Physics C: Solid State Physics* **18**, 3327 (1985).
[10] Buslaps, T., Johnson, R. L. & Jungk, G. Spectroscopic ellipsometry on 1T-$TiSe_2$. *Thin Solid Films* **234**, 549–552 (1993).
[11] Bertrand, Y., Leveque, G., Raisin, C. & Levy, F. Optical properties of $SnSe_2$ and SnS_2. *Journal of Physics C: Solid State Physics* **12**, 2907 (1979).
[12] Beal, a. R., Liang, W. Y. & Hughes, H. P. Kramers-Kronig analysis of the reflectivity spectra of 3R-WS2 and 2H-WSe2. *Journal of Physics C* **89**, 2449 (1976).
